# Genome integrity sensing by the broad-spectrum Hachiman antiphage defense complex

**DOI:** 10.1016/j.cell.2024.09.020

**Published:** 2024-10-11

**Authors:** Owen T. Tuck, Benjamin A. Adler, Emily G. Armbruster, Arushi Lahiri, Jason J. Hu, Julia Zhou, Joe Pogliano, Jennifer A. Doudna

**Affiliations:** 1 Department of Chemistry, University of California, Berkeley, Berkeley, CA 94720, USA; 2 Innovative Genomics Institute, University of California, Berkeley, Berkeley, CA 94720, USA; 3 California Institute for Quantitative Biosciences (QB3), University of California, Berkeley, Berkeley, CA 94720, USA; 4 School of Biological Sciences, University of California, San Diego, La Jolla, CA 92093, USA; 5 Department of Molecular and Cell Biology, University of California, Berkeley, Berkeley, CA 94720, USA; 6 Howard Hughes Medical Institute, University of California, Berkeley, Berkeley, CA 94720, USA; 7 MBIB Division, Lawrence Berkeley National Laboratory, Berkeley, CA 94720, USA; 8 Gladstone Institutes, University of California, San Francisco, San Francisco, CA 94720, USA; 9 Department of Bioengineering, University of California, Berkeley, Berkeley, CA 94720, USA

## Abstract

Hachiman is a broad-spectrum antiphage defense system of unknown function. We show here that Hachiman is a heterodimeric nuclease-helicase complex, HamAB. HamA, previously a protein of unknown function, is the effector nuclease. HamB is the sensor helicase. HamB constrains HamA activity during surveillance of intact double-stranded DNA (dsDNA). When the HamAB complex detects DNA damage, HamB helicase activity activates HamA, unleashing nuclease activity. Hachiman activation degrades all DNA in the cell, creating “phantom” cells devoid of both phage and host DNA. We demonstrate Hachiman activation in the absence of phage by treatment with DNA-damaging agents, suggesting that Hachiman responds to aberrant DNA states. Phylogenetic similarities between the Hachiman helicase and enzymes from eukaryotes and archaea suggest deep functional symmetries with other important helicases across domains of life.

## In brief

Hachiman is a nuclease-helicase complex that protects against bacteriophage infection by destroying both viral and host genomes.

## Graphical Abstract

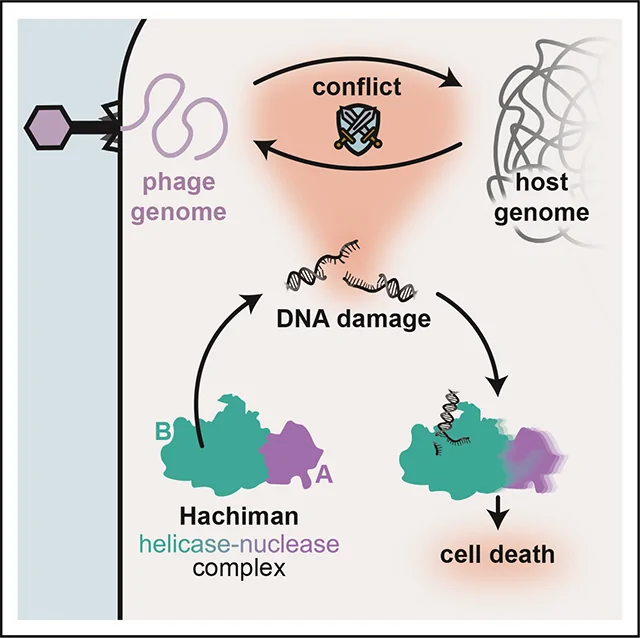

## INTRODUCTION

**Figure 1.**
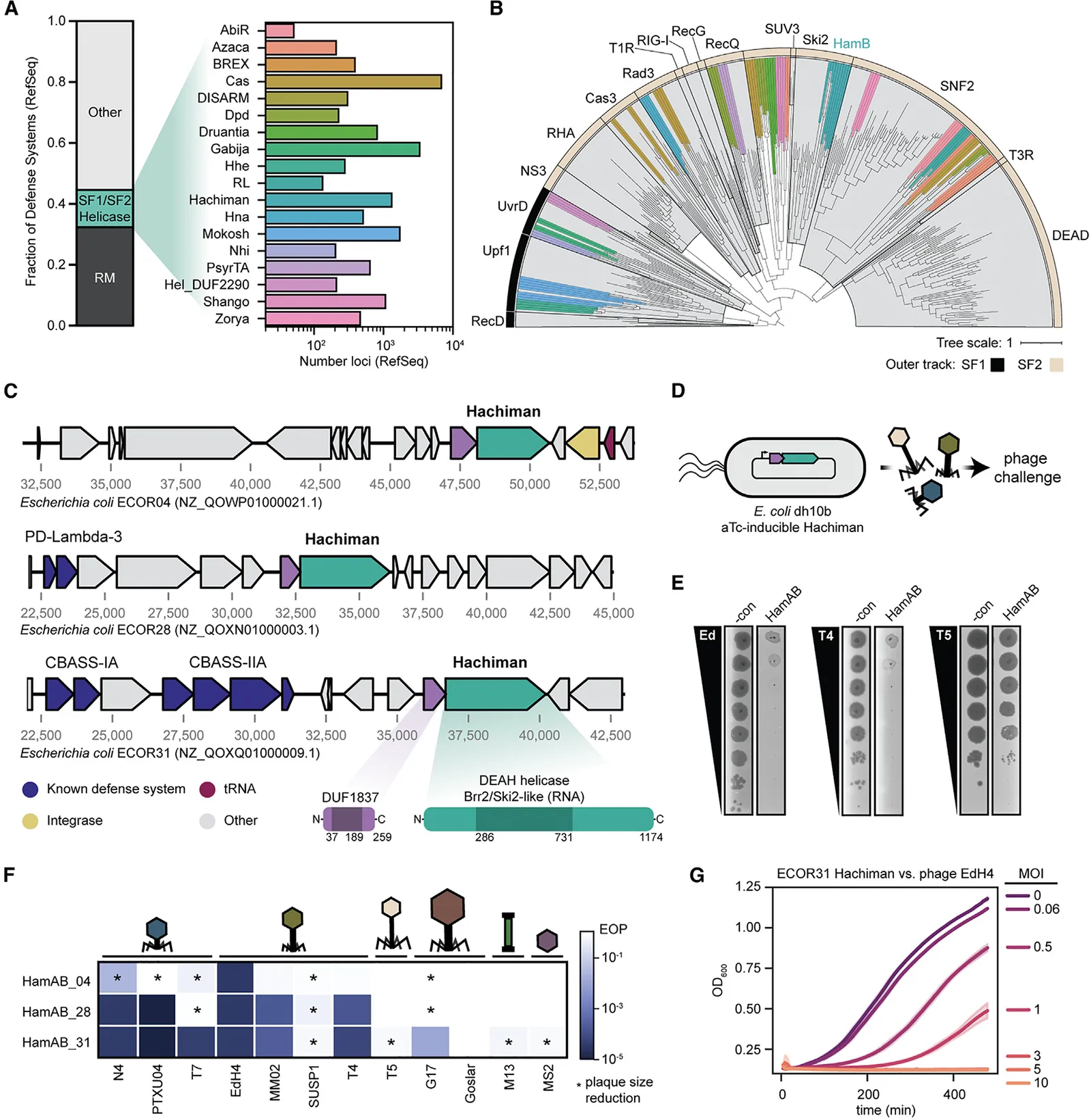
Hachiman is a two-component defense system that protects against diverse bacteriophages (A) Overview of SF1/SF2 helicase-containing phage defense systems found in RefSeq genomes in the DefenseFinder database.^17^ (B) Phylogenetic tree of core helicase domains of 329 helicases from defense systems from (A) and representative SF1/SF2 helicases.^12^ Helicase superfamily is provided in the outer track (SF1 in black, SF2 in tan) and representative families demarcated in gray clades with labels. Defense-system-associated helicases are colored as shown in (A). Details on tree construction and sequence alignment provided in STAR Methods. (C) Hachiman loci from *E. coli* strains ECOR04, ECOR28, and ECOR31 tested in this study. HamA genes are shown in purple and HamB genes shown in green. Additional defense systems identified in PADLOC^18^ are shown in blue, integrases in yellow, and tRNA genes in red. All other genes are shown in gray. (D) Overview of phage-defense assays. Native Hachiman loci are cloned under an anhydrotetracycline (aTc)-inducible promoter, pTet, and monitored for protection against diverse phages. (E) Representative plaque assays for ECOR31 HamAB against sensitive phages EdH4 and T4, as well as resistant phage T5. Data are presented as the mean of three biological replicates. (F) Comparison of different Hachiman loci against 12 diverse phages representing 12 unique phage genera. Data shown represent the mean of three biological replicates. Plaque assays without EOP reductions, but a measurable difference in plaque size are denoted with an asterisk. (G) Protection against phage EdH4 is complete at low MOI, but insufficient at high MOI. For (E)–(G), Hachiman is induced at 20 nM aTc and for (E) and (F) dCas13d targeting RFP is provided as a negative control. For (G), a negative control is shown in Figure S2D. Data are presented as the mean of three biological replicates ± standard deviation. See also Figures S1 and S2.

Helicases participate in innate and adaptive immune systems by “sensing” pathogen-associated molecular patterns (PAMPs).^1–13^ Many recently discovered antiviral defense systems in prokaryotes encode helicases homologous to diverse immune and regulatory helicases in eukaryotes (Figures 1A and 1B).^14–16^ One such system is Hachiman, a two-gene locus encoding HamA (a protein of unknown function, DUF1837) and the superfamily 2 (SF2) Ski2-like helicase HamB. Although present in >5% of prokaryotic genomes and capable of robust protection against phylogenetically distinct phages,^14,17^ molecular mechanisms governing Hachiman and many related helicase-containing immune systems remain unknown.

Here, we show that despite its homology to RNA helicases, HamB is a DNA helicase that activates DNase activity of HamA upon detection of damaged DNA. Cryogenic electron microscopy (cryo-EM) structures show how the HamAB complex binds DNA in different modes to facilitate immunity. Helicase “ratcheting” by HamB upon substrate recognition modulates the HamAB interface, leading to HamA activation and indiscriminate degradation of DNA. *In situ* fluorescence microscopy shows that Hachiman clears both host and phage DNA simultaneously, creating phantom cells devoid of genetic material. The observation of Hachiman activation in the absence of bacteriophage but in the presence of DNA-damaging agents suggests that Hachiman responds to DNA damage that accumulates during cell stress. Biochemical and structural data imply that ATP-bound HamAB contacts intact DNA, enabling detection of genome integrity and activation of the HamA effector when DNA damage surpasses a normal threshold. HamA nuclease activity may create additional sites for Hachiman binding and activation, leading to amplification of the immune signal and culminating in restriction of phylogenetically diverse phages.

HamB helicase domain organization and its ability to regulate the HamA effector enables controlled activation that may be a principle of other helicase-containing defense pathways. Like Hachiman, other defense systems may act in response to cell stressors, including, but not limited to, phage infection.

## RESULTS

### Hachiman confers broad-spectrum protection against diverse bacteriophages

Helicases are common components of immune systems in eukaryotes.^9,19^ This is also true for prokaryotic immune systems. Of the ~150 prokaryotic defense systems cataloged in DefenseFinder,^17^ 18 contain an SF1/SF2 helicase, comprising nearly 20% of non-restriction-modification (RM) defense loci identified in RefSeq (Figure 1A).^20^ Using 95 defense system helicases and 236 well-characterized representative SF1/SF2 helicases,^12^ we performed a phylogenetic analysis of the core helicase domain (Figure 1B; see STAR Methods). We assigned 20 of the 25 helicases to an established helicase subfamily, spanning 7 subfamilies. Helicases from Shango (SngC), BREX (BrxHI), Druantia (DruE), and Dpd (DpdJ) formed a potentially distinct clade of SF2 helicase family defined by antiphage immune functions. A helicase from DISARM (DrmA) also could not be confidently assigned to a known helicase family. The Hachiman-encoded HamB protein is closely related to SF2 Ski2 helicases, orthologs of which have diverse activities on RNA and DNA substrates (Figures 1B and S1).^21,22^

To establish a cell-based assay for assessing Hachiman function, we identified distinct Hachiman loci in *E. coli* strains ECOR04, ECOR28, and ECOR31 using PADLOC (Figures 1C, S2A, and S2B).^18,23^ We challenged cells expressing Hachiman in plaque assays using *E. coli* phages representing twelve distinct genera (Figures 1D, 1E, and S2C; Key resources table).^24^ HamAB from ECOR31 conferred the greatest degree of defense, providing 10^2^- to 10^5^-fold reduction in efficiency of plaquing for eight diverse double-stranded DNA (dsDNA) phages (Figures 1F and S2C), consistent with the broad-spectrum activity of *Bacillus cereus* Hachiman against *Bacillus subtilis* phages.^14^ Hachiman conferred near-complete defense against sensitive phages at low multiplicity of infection (MOI < 1), but diminished protection at high viral doses (MOI > 1) (Figures 1G and S2D–S2F). We confirmed that Hachiman limits the production of new phage particles (Figures S2E and S2F). At low MOI, bacterial growth is unaffected, whereas at high MOI, no growth is observed (Figure S2D). Nonetheless, phage production remains limited-to-nonexistent during Hachiman-mediated defense (Figures S2E and S2F), meaning that the interaction between Hachiman and phage leads to cell death and restriction of phage progeny. These data imply that Hachiman functions by abortive infection (Abi), a type of programmed cell death in which infected cells sacrifice themselves before phage infection matures, preventing viral spread.^25^ To control for artifacts arising from overexpression, we also confirmed that ECOR31 Hachiman defends against phage while under the control of its native promoter and on a low-copy plasmid (Figure S2G).

The four phages resistant to tested Hachiman systems (T5, MS2, M13, and Goslar) possess unique genome properties. MS2 is an ssRNA phage and lacks a dsDNA genome, whereas M13 uses rolling-circle replication to produce its single-stranded DNA (ssDNA) genome.^26^ The dsDNA genomes of T5 and Goslar have limited accessibility to defense systems as they are compartmentalized before and during infection, respectively.^27–29^ Overall, phage-challenge experiments suggest that Hachiman activity protects against diverse dsDNA phages recognizing and subverting a central feature of dsDNA phage infection.

### Structural basis of HamAB complexation

**Figure 2.**
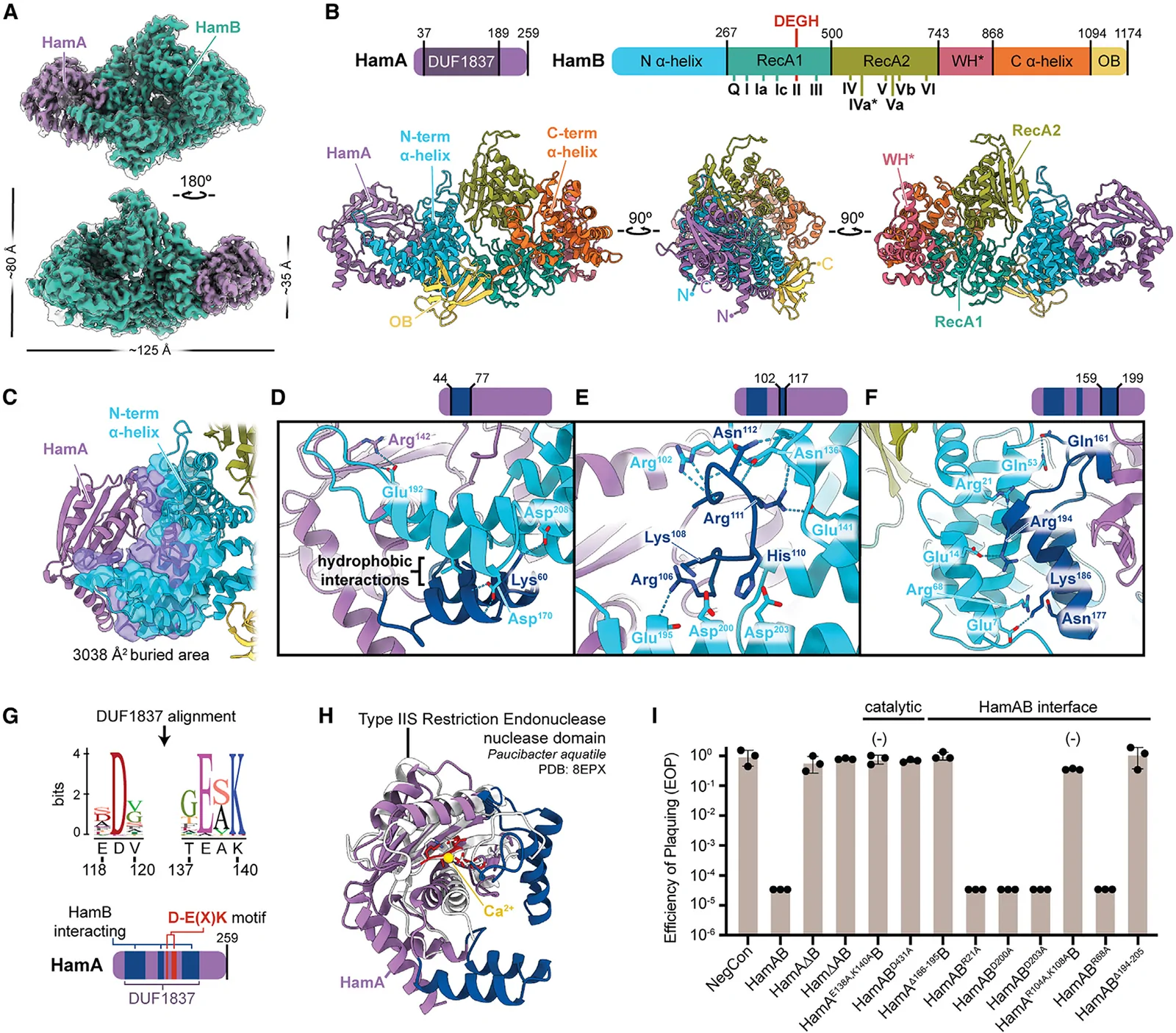
Structural basis of Hachiman complexation and identification of the HamA active site (A) Cryo-EM density of the *E. coli* ECOR31 apo HamAB complex. The sharpened map is colored, whereas the unsharpened map is overlaid and transparent. (B) Orthogonal views of the HamAB structure, with domains colored according to the key above. Walker motifs are annotated in the HamB RecA1 and RecA2 domains. (C) Overview of the HamA-HamB NAH interface, with surfaces involved in the interaction shown. (D–F) Detail of three subregions, HamA^30–63^ (D), HamA^102–117^ (E), and HamA^159–199^ (F), contributing to the AB interface. Residues contributing to hydrogen bonding interactions are shown as sticks and are labeled with colors corresponding to the key above each view and in (B). (G) Sequence logo resulting from alignment of HamA DUF1837 ORFs. The ECOR31 HamA sequence and corresponding positions are shown below each residue logo. (H) Structural superimposition of the nuclease domain from the *P. aquatile* type IIS restriction modification system with HamA. (I) Plaque assays demonstrating the ability of HamAB and various mutants to confer defense against phage EdH4. Individual data points of three independent biological replications are shown along with the mean and standard deviation. The (−) symbol indicates a reduction in plaque size. See also Figure S3.

To determine the molecular basis of Hachiman function, we purified HamA and HamB individually. Only HamB was soluble in isolation. Coexpression of the complete native Hachiman locus produced a complex of HamA and HamB (Figures S3A and S3B). A 2.7-Å cryo-EM structure of the isolated HamAB complex is a 1:1 heterodimer (HamA_1_:HamB_1_; Figures 2A and S3A–S3G; Table S1). The domain organization of HamB is generally consistent with Ski2/Brr2 helicases, with two stacked RecA-like helicase domains, RecA1 and RecA2, comprising the helicase core.^30^ A degenerated winged-helix domain (WH*) and C-terminal α-helical region (CAH, C α-helix) form the likely nucleic-acid-binding cleft (Figure 2B). At the N terminus, an α-helical bundle (NAH, N α-helical) common to HamB orthologs, but not found in related Ski2 helicases, contributes to binding HamA. At the C terminus, a barrel-like fold reminiscent of oligonucleotide-binding (OB) domains sits on the side of the complex. HamB folds with intact helicase motifs, including active site DEGH residues (Figures S3H and S3I).

The apo HamAB structure shows how HamA contacts the HamB NAH, with three AB interface regions contributing to 3,038 Å^2^ of total buried surface area (Figures 2C and S3J). The first subregion contains a helix-loop-helix, which stacks with a HamA helix-loop-helix in the reverse orientation (HamA^30–63^; Figure 2D). The second region is in the center of the interface and includes numerous hydrogen bonds between HamB and an extended HamA-interacting loop (HamA^102–117^; Figure 2E). The third subregion on the HamB N-terminal side is another instance of helical docking with predicted hydrogen bonding and nonpolar interactions (HamA^159–199^; Figure 2F). Structural and biochemical analyses of the AB interface suggest that HamB solubilizes HamA (Figures 2C–2F, S3A, and S3B).

### HamA DUF1837 encodes a nuclease

The role of HamA is unknown. Alignment of HamA sequences revealed a highly conserved D-E(X)K motif consistent with metal ion-dependent phosphodiester hydrolysis (Figure 2G).^31^ Structurally, HamA is most similar to the type IIS restriction endonuclease from *Paucibacter aquatile*,^32^ with conservation of the core helix/sheet motif (Figures 2H and S3K). HamA diverges from the *P. aquatile* nuclease in regions of DUF1837 that facilitate binding to HamB (Figures 2C–2H).

Cell-based phage defense assays showed that deletion of HamA or HamB, or mutation of their putative active site residues to create HamA^E138A,K140A^B (HamA*B, nuclease-deficient) or HamAB^D431A^ (HamAB*, helicase-deficient), ablated defense (Figure 2I). Although single-interface mutations failed to impact defense, double mutation of a conserved interface motif (R/K) XX(R/K), or deletion of entire helix-loop-helix motifs in HamA, blocked Hachiman function. However, these cells were still viable, suggesting that HamA must be activated by HamB to trigger effector function. Together, these results show that Hachiman requires both nuclease (HamA) and helicase (HamB) activities for function and that complexation of HamA and HamB is essential for activation of phage defense.

### HamB is a DNA helicase

**Figure 3.**
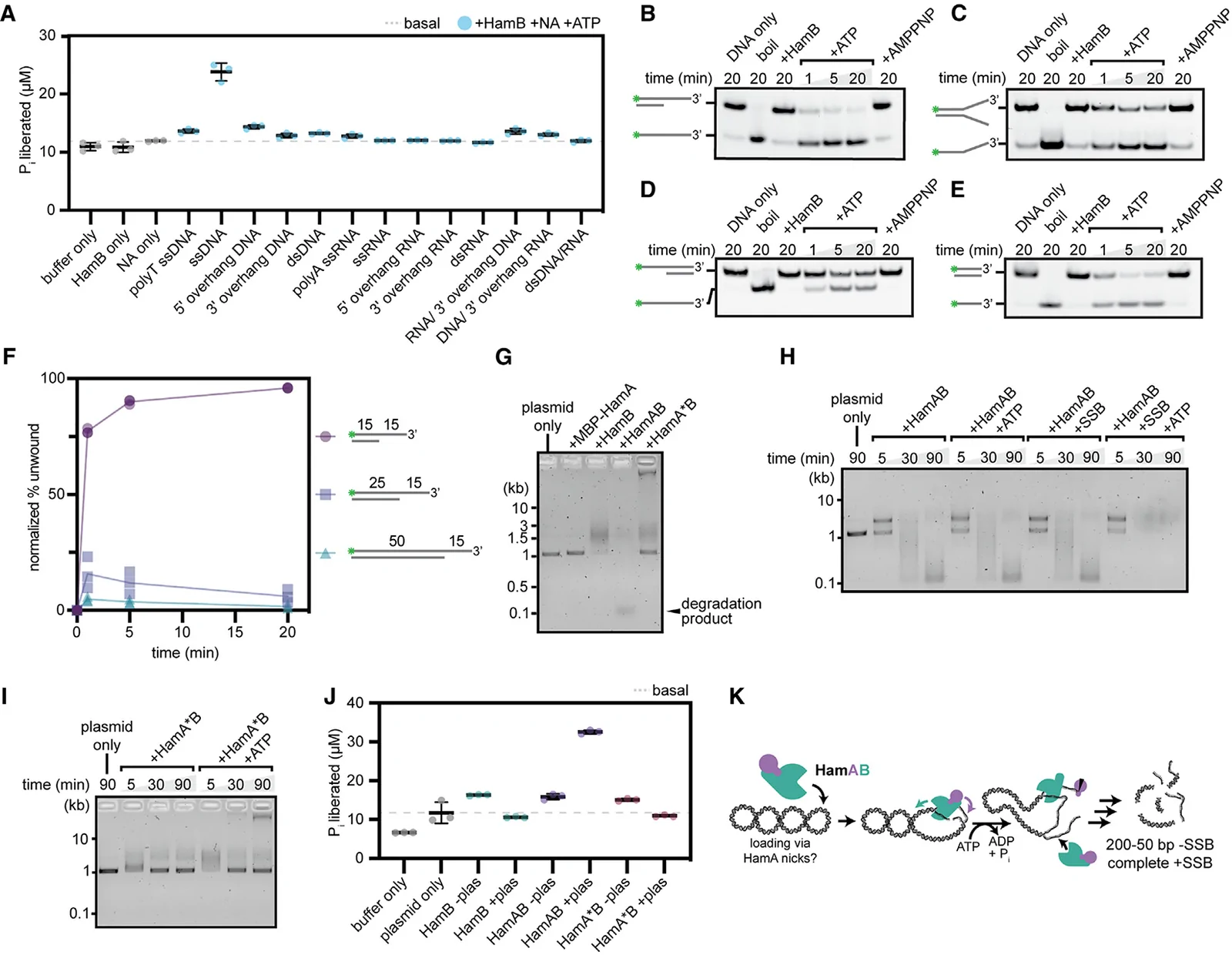
HamAB is a DNA nuclease/helicase that degrades plasmids *in vitro* (A) Malachite green ATPase assays of HamB against a panel of nucleic acid substrates. Individual data points of three independent biological replicates and the mean and standard deviation are shown. (B–E) HamB DNA unwinding assays on substrates with a 15-bp duplex and a 15-nt 3′ OH (B), forked 15-nt OH (C), 15-nt 5′ OH (D), and no overhang (E). DNA substrates are labeled with 5′ FAM. Gels are representative of three independent biological replicates. (F) Normalized percent unwinding of DNA substrates with 15 bp (circles), 25 bp (squares), and 50 bp (triangles) duplex lengths, all labeled with 5′ FAM and with a 15-nt 3′ OH. Individual data points shown are quantifications of replications of unwinding assays in the format of (B)–(E) normalized against basal unwinding (see STAR Methods). (G) *In vitro* plasmid clearance assay after 90 min at 37°C with ATP using MBP-HamA, HamB, HamAB, and HamA*B visualized on a 0.75% agarose gel. (H) Time course of HamAB plasmid clearance with addition of ATP or *E. coli* SSB, visualized on a native agarose gel. (I) Time course assay as in (H) with mutant HamA*B. (J) ATPase activity of HamB, HamAB, and HamA*B, with or without supercoiled plasmid substrates. Individual data points of three independent biological replicates and the mean and standard deviation are shown. (K) Cartoon depicting a model for HamAB-mediated plasmid degradation. See also Figure S4.

Despite its functional requirement for phage defense, the identity of the HamB helicase substrate is unclear. Using a malachite green assay that detects orthophosphate release during NTP hydrolysis,^33^ we found strong HamB ATPase activity in the presence of ssDNA (Figure 3A). To test HamB DNA helicase activity, we performed DNA unwinding assays by incubating HamB with DNA duplexes of varying lengths and single-stranded overhangs. HamB was capable of ATP-dependent unwinding of a 15-bp duplex with a 15-nt 3′ overhang (Figure 3B). In addition, HamB unwinds forked, 5′ overhang and blunt DNA duplexes, albeit with lower efficiency compared with 3′ overhang-containing substrates, suggesting promiscuous substrate acceptance (Figures 3C–3E and S4A–S4F). Longer duplex lengths are not well tolerated (Figure 3F). Testing of different DNA duplex and overhang lengths showed that HamB processes a range of DNA substrates but prefers longer 3′ overhangs (Figures S4A–S4F).

### HamAB degrades plasmids *in vitro*

HamB unwinds DNA substrates, and HamA is a putative nuclease. To determine whether HamA cuts DNA, and to ascertain the combined functions of the HamAB complex, we tested activity against purified plasmid DNA. Titration of the wild-type (WT) HamAB complex, but not HamA*B, in reactions with supercoiled plasmid DNA show initial plasmid nicking followed by a ladder of degradation products, which converged to sizes between 50 and 200 bp, irrespective of input plasmid topology (Figures 3G, S4G, and S4H). We also observed cleavage of short dsDNA and ssDNA substrates (Figures S4I and S4J). Our observations are consistent with HamA acting as a nuclease effector in Hachiman immunity, though the exact nature of HamA cleavage remains unclear owing to its insolubility *in vitro* (Figure S3A). Although HamA*B cannot cleave DNA, it forms a low-mobility species upon addition of ATP, which may represent a different state captured only when the HamA nuclease is catalytically deactivated (Figure 3G).

To assess the possible influence of phage-encoded single-stranded binding (SSB) protein, which has been implicated in activating Hachiman and other antiphage defense systems,^34–36^ we incubated reactions with either *E. coli* SSB (EcSSB) or phage T4 gp32 (T4SSB) prior to addition of Hachiman components. Both types of SSB induced complete ATP-dependent plasmid degradation, arguing against direct recognition of phage SSB in Hachiman activation (Figures 3H, S4K, and S4L). In time-course experiments, addition of either *Ec*SSB or T4SSB accelerated the rate of plasmid interference (Figures S4K and S4L). SSB also facilitates complete ATP-dependent degradation of a 75-bp dsDNA, though ssDNA substrates are protected by excess SSB (Figures S4I and S4J). *Ec*SSB did not have an observable effect in HamA*B time courses (Figure S4M), implying that HamA nuclease activity is required for subsequent DNA unwinding *in vitro* (Figure 3I). SSB stimulates HamAB DNA unwinding and cleavage independent of SSB type, potentially by preventing reannealing of nascent ssDNA (Figure 3H).^37^

We noted that the low mobility species observed in HamA*B-plasmid reactions accumulate in an ATP-dependent manner (Figure 3I). ATPase assays with HamB, HamAB, and HamA*B in the presence of plasmid DNA reveal that, unlike HamAB, both HamB and HamA*B suppress ATPase activity upon substrate addition (Figure 3J). The low mobility species may therefore represent a state in which HamA*B ATP-binding enables association with, but not cleavage of, intact dsDNA. These data imply not only that plasmid destruction requires DNA cleavage by HamA but also that this activity may be coupled with HamB ATPase activity (Figure 3J). Considering that HamAB does not require ATP to degrade plasmid DNA in the absence of SSB, we postulate that HamAB loads DNA ends induced by HamA nicks generated *in vitro* by high relative concentrations of complex (Figure S4G). Nicking triggers ATP hydrolysis, which, in turn, activates further HamA-mediated degradation (Figure 3K). When the HamA nuclease is inactivated, HamB does not load DNA ends but can nonetheless bind intact DNA in an ATP-dependent manner (Figure 3J).

### Structural basis of HamB DNA binding

**Figure 4.**
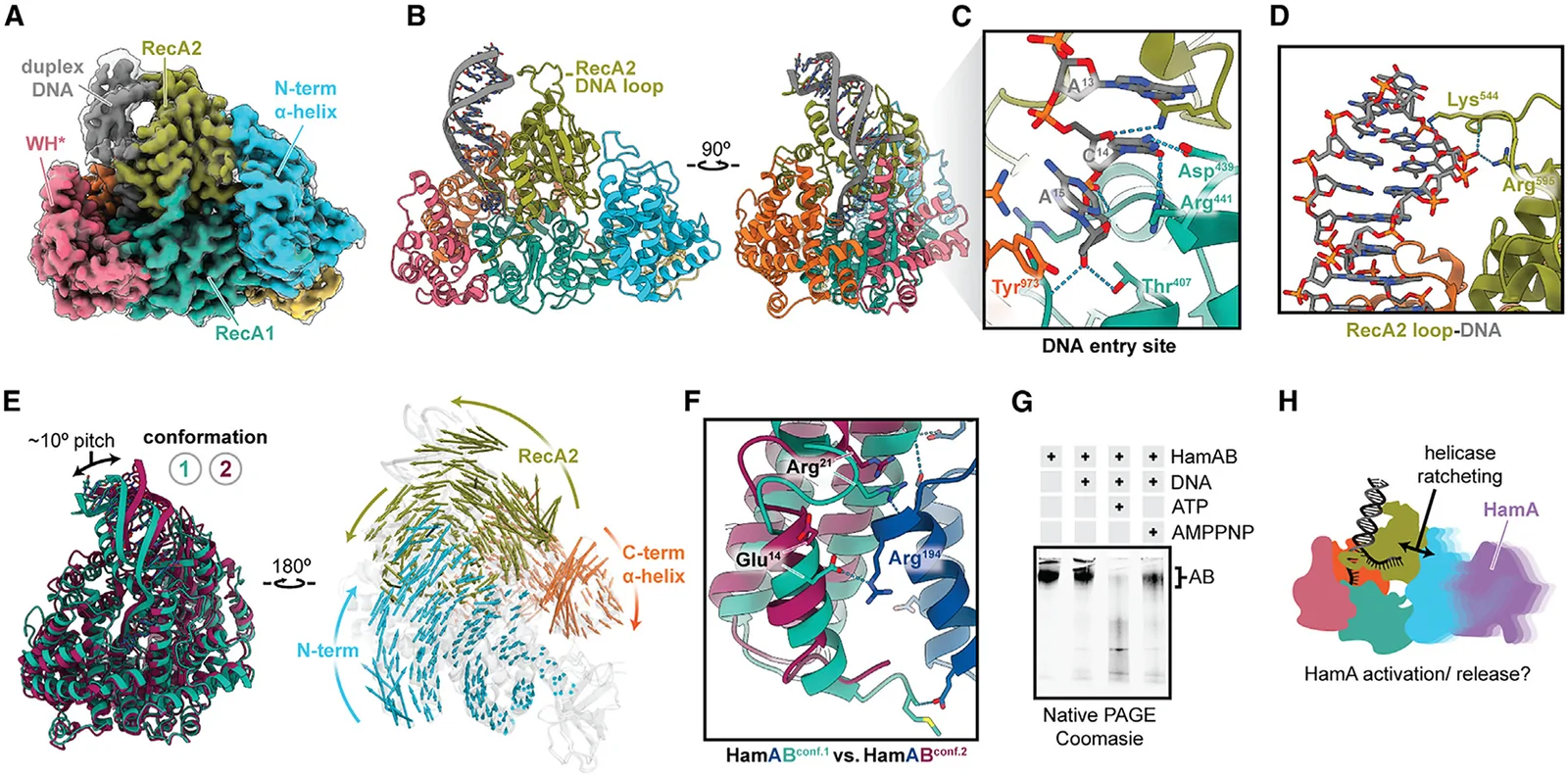
Structural basis of HamB-DNA binding and helicase ratcheting (A) Cryo-EM density of the 2.8-Å HamB-DNA density. The sharpened map is colored according to domain, whereas the unsharpened map is overlaid and transparent. (B) Orthogonal views of the 2.8-Å HamB-DNA structure. (C) Detail of the 3′ end of the DNA buried within the DNA entry site of HamB. Hydrogen bonds and contributing residues are shown with a dashed line. (D) Detail of the DNA duplex-interacting RecA2 loop. (E) Left, superimposed conformers of HamB-DNA viewed from the DNA side, with conformation 1 (2.8 Å) colored teal and conformation 2 (2.9 Å) colored burgundy. Right, conformations 1 and 2 viewed from the NAH side and transparent, with vectors colored according to domain representing motion between the two conformations. Vectors are scaled 2× and are calculated using modevectors. (F) Representative disruption of the predicted AB interface between the two HamB conformations. AB interactions disrupted by HamB motion are shown and labeled. (G) Native PAGE of reactions of the HamAB complex with the DNA where ratcheting was observed in cryo-EM. ATP and DNA appear to dissociate the AB complex. (H) Model for HamB signal transduction to the NAH and concomitant release of HamA. See also Figure S5.

Our biochemical studies suggest two modes of DNA binding, one that triggers ATP hydrolysis and cleavage (Figure 3I) and one that enables binding of HamAB to intact dsDNA (Figure 3J). We observed ATPase activity upon incubation of HamB with a mixed base ssDNA substrate. We performed cryo-EM analysis on a complex of HamB and ssDNA with ATP added during complexation. In the resulting 2.8 Å reconstruction, HamB retains the general domain organization observed in the apo HamAB structure (Figures 4A, 4B, and S5A–S5I; Table S1) but lacks the extended helix-loop-helix motif that contributes to HamA binding (Figures 2D and 4B). Disorder of this region in the HamB-DNA structure further supports its important role in complexation. Surprisingly, despite the addition of only ssDNA to HamB during sample preparation, we observed duplex DNA in the cryo-EM density. The duplex, which is 8 bp in length, arises from a partially palindromic region of the DNA substrate. The duplex appears partially unwound, with one 3′ end bound within the HamB nucleic-acid-binding pocket (Figure 4C). Several residues, including, but not limited to, canonical Walker motifs, contribute to ssDNA binding in the helicase core (Figures 4D, S3H, and S3I). In the midsection of the duplex, a “pin” reminiscent of the strand unwinding wedge in the PriA primosomal helicase lies in the center of the duplex and pries the strands apart by pi-stacking and physical occlusion (Figure S5J).^38,39^ Observation of a 3′ end in the entry site of the helicase is consistent with the 3′ to 5′ polarity determined *in vitro* (Figure 3B). When we mutagenized a conserved threonine, which forms a hydrogen bond with the 3′ hydroxyl (Figure 4C), Hachiman lost antiphage activity (Figure S5K). Recognition of a 3′ DNA end is important for immune activation.

### Helicase ratcheting may activate HamAB

We noticed significant conformational variability in the HamB-DNA particle ensemble. Using three-dimensional (3D) variability analysis and 3D classifications, we resolved an alternative conformation (conformation 2) of HamB to a nominal resolution of 2.9 Å (Figures S5A–S5I; Table S1; STAR Methods). In the alternate conformation, we observe repositioning of the RecA2, NAH, and CAH domains, coupled with pitching of the DNA duplex by approximately 10 (Figure 4E, left). DNA contacts and the RecA1, WH*, and OB folds remain virtually unchanged. Viewed from the HamA direction, the NAH and CAH rotate clockwise, whereas RecA2 moves counterclockwise (Figure 4E, right). Large-scale movement of the NAH-RecA2 interface involves remodeling of interface regions. For example, Tyr^525^ and Trp^521^ residues on a distal RecA2 sheet contacting the NAH shift ~9 Å between the two conformations (Figure S5L). We propose that dynamic switching between HamB conformations represents helicase ratcheting upon entry of the DNA substrate into the active site and triggering of ATPase activity. Motion of the RecA2 domain upon helicase ratcheting transduces to the NAH (Figure 4E). The NAH is responsible for binding the HamA nuclease (Figures 2C–2F and 2I). We superimposed each conformation of HamB bound to DNA with HamB in the apo complex structure. In conformation 1, HamB is in approximately the same position as HamB in the AB complex. Changes in conformation 2 appear to disrupt predicted interactions with HamA (Figure 4F), including hydrogen bonds in HamA^159–199^, a region shown to be essential for defense (Figure 2I).

Structural evidence suggests that helicase ratcheting transduces motion to the NAH. To test whether helicase motions disrupt the complex, we incubated HamAB with the mixed base ssDNA seen in HamB-DNA structures. Addition of ATP and DNA modulates the HamAB complex, suggesting that ATP hydrolysis upon substrate recognition provides input energy to allosterically activate HamAB, potentially by releasing HamA (Figure 4G). We observed ATP-dependent disassembly of the complex via size exclusion chromatography (Figure S5M). Together, these data support a model in which structural changes in HamB may release HamA upon entrance of ssDNA into the helicase active site (Figure 4H). In further support of this model, plasmid assays show that nicked DNA triggers ATPase activity, whereas intact DNA untouched by HamA nucleolytic activity does not activate ATP hydrolysis (Figure 3J).

### Hachiman degrades phage and host DNA simultaneously during infection

**Figure 5.**
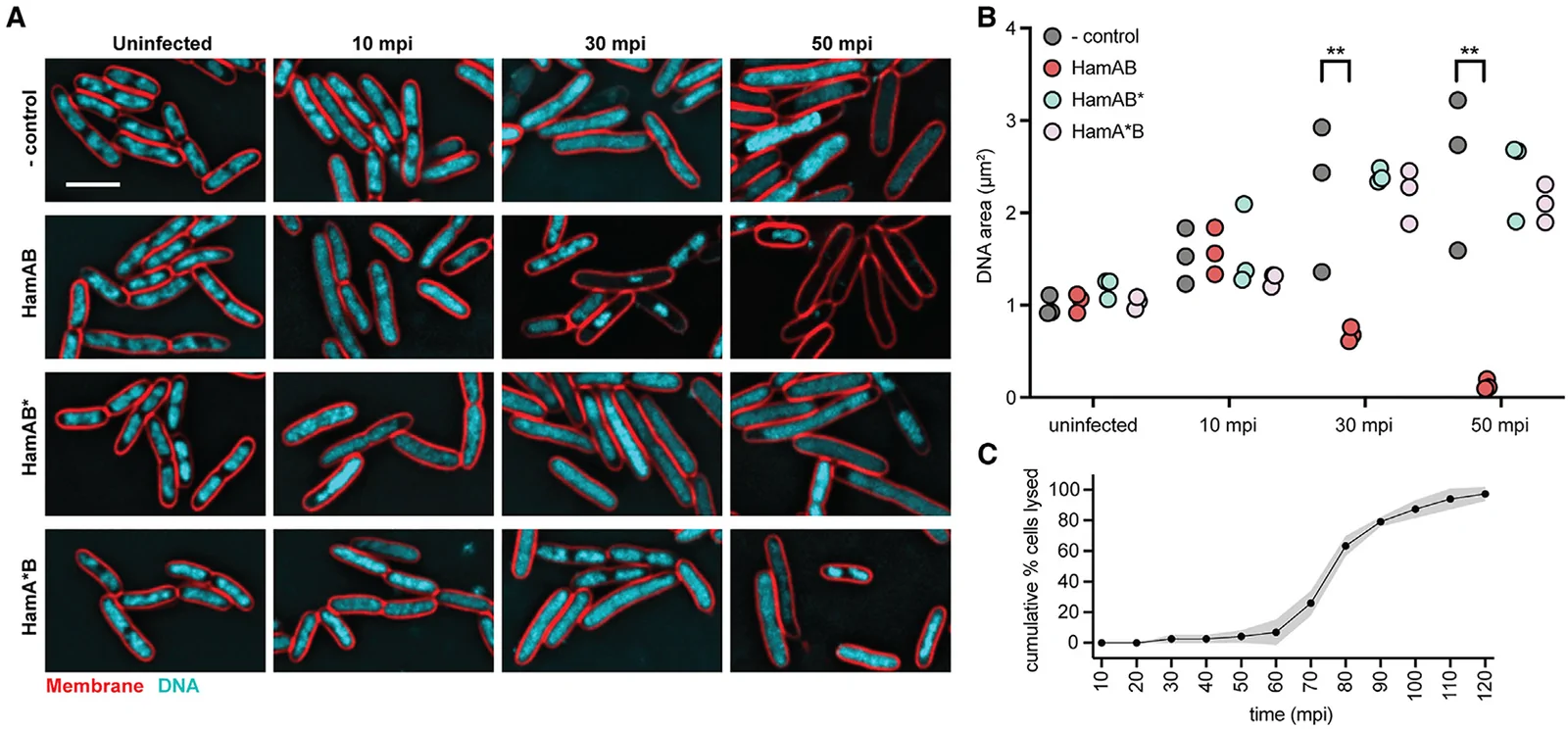
Hachiman defends against bacteriophage by nonspecific DNA clearance (A) EdH4 infection time course in *E. coli* expressing wild-type HamAB, HamAB* (HamAB^D431A^), or HamA*B (HamA^E138A,K140A^B) or lacking the Hachiman system (control). Cell membranes were stained with FM4–64 (red) and DNA was stained with DAPI (cyan). Scale bar, 3 μm. MOI ≈ 2. (B) Quantification of intracellular DAPI-stained DNA cross-sectional area over the course of EdH4 infection. Dots represent individual medians from three biological replicates. ** *p* < 0.01 by Dunnett’s test. *n* >225 in total across all replicates for each condition (see STAR Methods). (C) Time-to-lysis of EdH4 infecting the control strain based on time-lapse bright-field microscopy under the same growth and infection conditions as the time course in (A) and (B). Black points represent the mean cumulative percentage of total lysed cells that have burst at 10 min intervals over the course of EdH4 infection, measured in triplicate. Shaded region represents the standard deviation.

Hachiman binds and degrades DNA. To connect the proposed HamAB structural states with cellular activities, we visualized Hachiman responding to phage infection *in vivo* using time-course fluorescence microscopy. In uninfected cells, neither WT nor inactivated HamAB affected nucleoid morphology (Figures 5A and 5B). When we challenged the control strain lacking Hachiman with sensitive phage EdH4 (Figure 1F), we observed that decondensed DNA begins to appear 10 min post infection (mpi; Figure 5A). There was no significant difference in the development of this phenotype compared with cells expressing inactive HamAB mutants (Figure 5B, HamA*B or HamBA*). However, when we infected cells expressing WT HamAB, the nucleoids significantly decreased in size by 30 mpi. By late infection (50 mpi), most cells are “phantom cells,” containing only a small punctum or no visible DNA (Figure 5B). Across three biological replicates, at 50 mpi, the median DNA cross-sectional area was <0.2 μm^2^ in our HamAB strain. In contrast, the median DNA cross-sectional area was >1.5 μm^2^ at 50 mpi in the absence of HamAB or during expression of an inactive mutant.

As determined by time-lapse bright-field microscopy, the average time-to-lysis for EdH4 infecting the control strain under our imaging conditions is ~75 mpi (Figure 5C). Hachiman is activated and degrades DNA well before host-cell lysis under WT conditions, preventing the release of phage progeny at the expense of cellular viability.^25^ These observations are consistent with the Abi phenotype observed in phage production assays (Figures 1G and S2D–S2F) and agree with biochemical and structural data identifying Hachiman as a DNA-degrading defense system.

### DNA damage activates Hachiman

Hachiman responds to phage infection by clearing cells of DNA. The HamB helicase recognizes 3′ ssDNA ends and activates ATP hydrolysis (Figures 2B–2F and 2J), which, in turn, activates the HamA nuclease (Figures 4E–4H). Because Hachiman defends against diverse bacteriophage genera with little or no protein homology, we considered the possibility that Hachiman does not directly recognize a conserved phage component such as phage-encoded SSB.^34^ Instead, Hachiman could sense general changes in host physiology.

**Figure 6.**
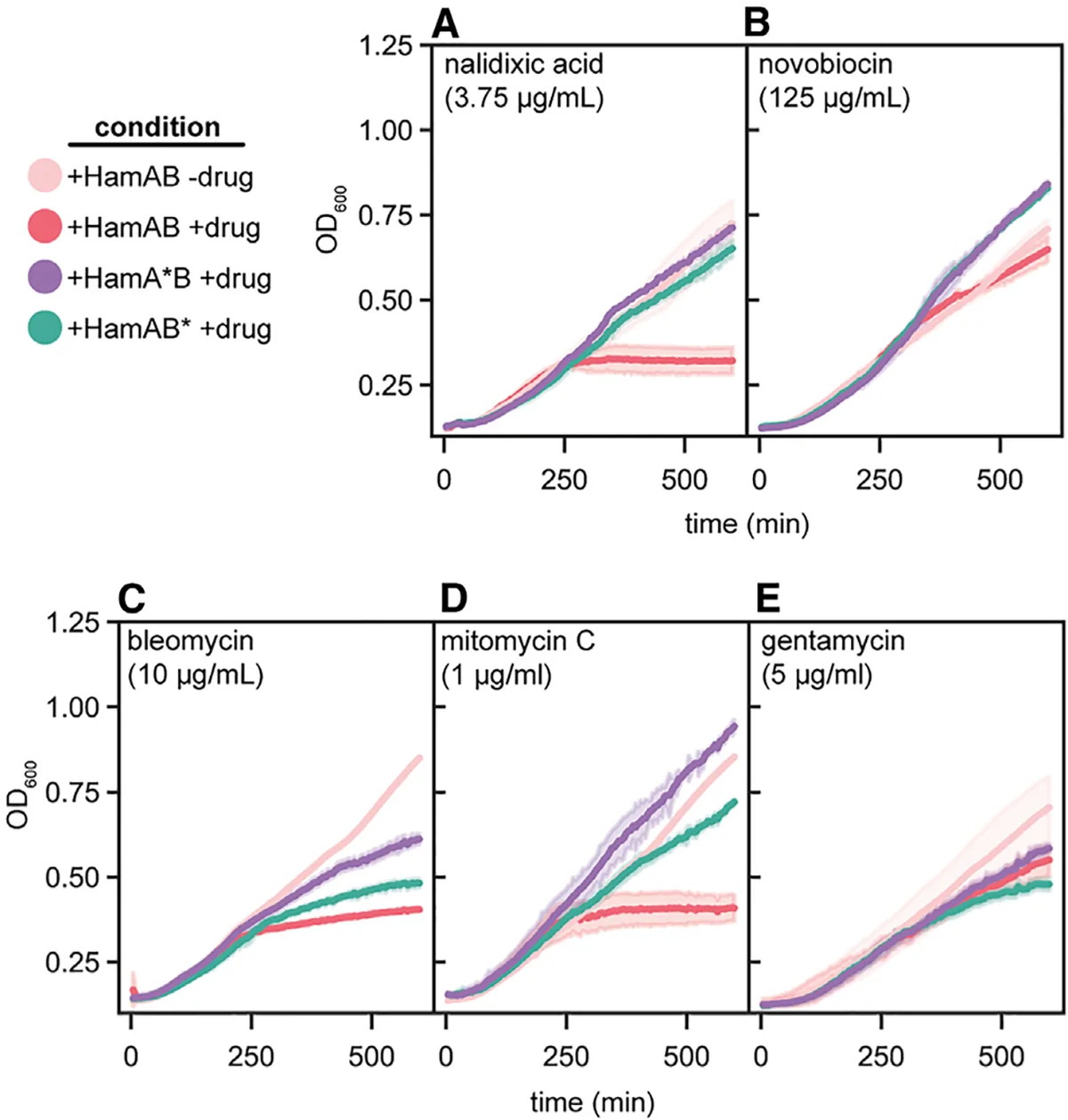
DNA damage activates Hachiman (A–E) Cell growth of *E. coli* MDS42 expressing wild-type HamAB, HamA*B, and HamAB* at 20 nM aTc in the absence or presence of minimum inhibitory concentrations of nalidixic acid (A), novobiocin (B), bleomycin (C), mitomycin C (D), and gentamycin (E). Growth curves are colored according to condition. See Figure S6 for complete minimum inhibitory concentration determinations. Data are presented as the mean of three biological replicates ± standard deviation. See also Figure S6.

We reasoned that small molecules that interfere with DNA metabolism might differentially engage Hachiman and elicit bacterial toxicity if it responds to changes in host genome integrity. To test this hypothesis, we treated cells expressing Hachiman with minimum inhibitory concentrations of DNA-damaging antibiotics. To control for confounding factors arising from potential drug-induced excision of endogenous MGEs, we used the *E. coli* MDS42 strain background, which is devoid of cryptic prophage and insertion sequence elements.^40,41^ We first showed that Hachiman retains antiphage activity in this strain background (Figures S6A and S6B), confirming that Hachiman activity is not dependent on known cryptic prophages and insertion sequence elements. We next treated cells with nalidixic acid (nal), a quinolone inhibitor of DNA gyrase and topoisomerase IV (topo IV).^42,43^ Aberrant persistence of the protein-DNA linkages during topoisomerase inhibition by nal results in DNA nicks, replication fork arrest, and double-strand breaks (DSBs).^44–47^ In the absence of bacteriophage, we observed growth inhibition in response to minimum inhibitory amounts of nal when WT Hachiman was present compared with HamA- and HamB-inactivated mutants (Figures 6A and S6C). Novobiocin (novo) is an aminocoumarin that also interferes with gyrase and topo IV, but by an orthogonal mechanism which subverts direct DNA damage.^48,49^ In growth experiments, WT HamAB had minimal differential effects after novo treatment (Figures 6B and S6D). Rather than inhibit an essential enzyme regulating DNA topology, the polyketide/peptide bleomycin directly induces ss- and dsDNA breaks by generation of radical intermediates.^50^ WT Hachiman caused elevated toxicity during bleomycin treatment compared with HamA and HamB mutants (Figures 6C and S6D). Cultures treated with mitomycin C, an alkylating agent that causes interstrand DNA crosslinks and subsequent DSBs, also enhanced toxicity of WT Hachiman compared with mutants (Figures 6D and S6E).^51^ In both mitomycin C and bleomycin treatment conditions, we noticed that strains expressing mutant HamB were consistently more sensitive than HamA mutants. As a control, we treated cultures with gentamycin, an aminoglycoside that inhibits translation by binding to the 30S ribosomal subunit.^52^ Consistent with DNA-damage-dependent activation, we observed near-equivalent responses to all Hachiman constructs to gentamycin exposure (Figures 6E and S6F). Our results demonstrate that Hachiman can reliably be triggered in the absence of bacteriophage, that activation follows direct DNA damage, and that activation requires the combined catalytic activities of HamA and HamB.

### Hachiman associates with intact dsDNA

**Figure 7.**
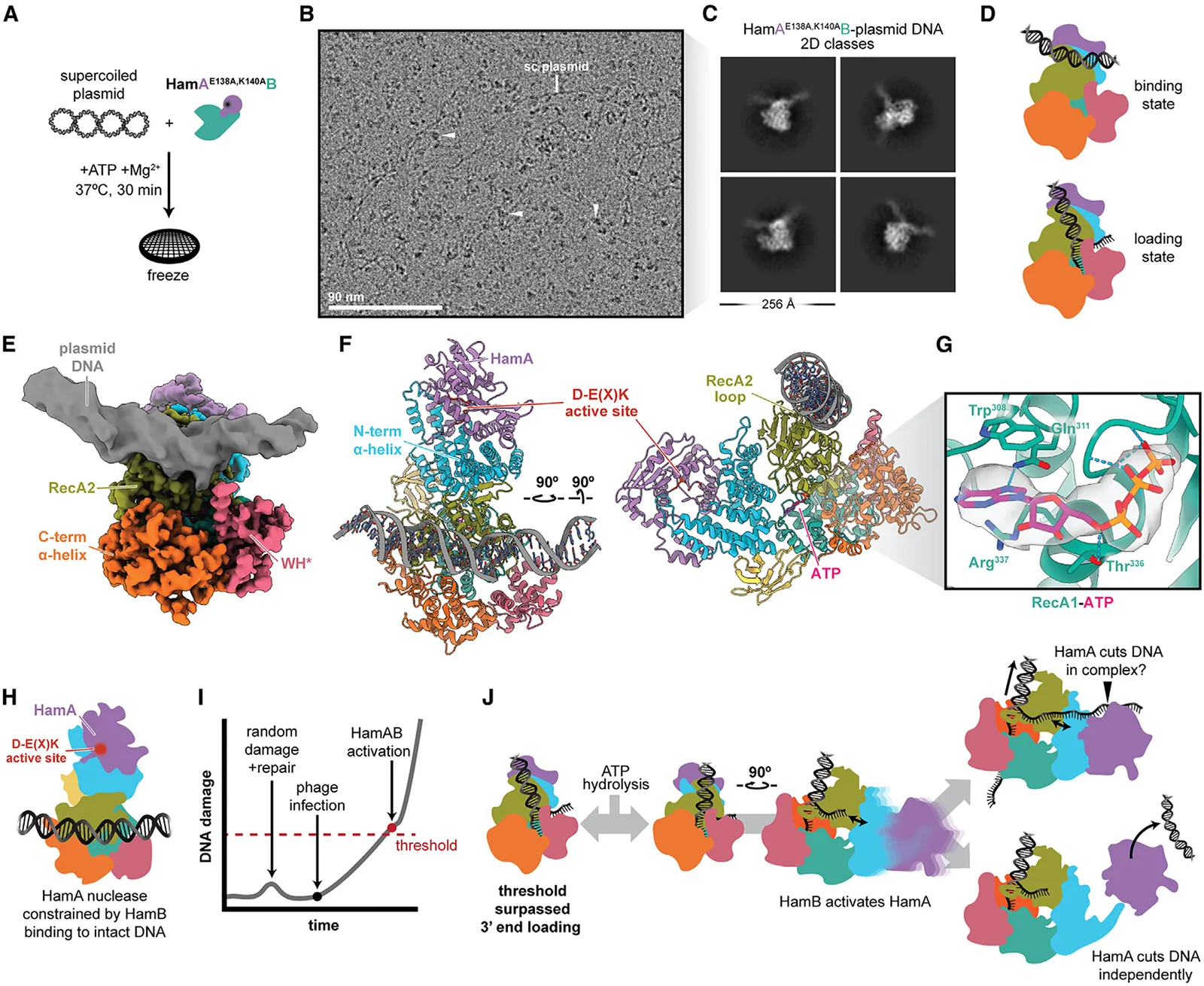
Hachiman scans intact dsDNA (A) HamA*B-plasmid +ATP specimen preparation. (B) Representative motion-corrected, dose-weighted cryo-EM micrograph from the HamA*B-plasmid DNA dataset. Plasmid DNA and bound particles are indicated with white arrows. (C) Representative 2D classes of particles bound to plasmid DNA. (D) Cartoon depicting the scanning state resolved here and comparison with the loading state resolved in the HamB-DNA dataset. (E) Composite cryo-EM density colored according to domain. Protein regions are from the 3.2-Å deepEMhancer-sharpened map, whereas DNA is from the 3.2-Å sharp map masked and B-factor refined to display helical features. (F) Orthogonal views of the HamA*B-plasmid DNA structure. The DNA sequence is unknown. (G) Detail of ATP in HamB, with residues and hydrogen bonds shown. The density is masked to ATP. (H) Cartoon showing separation of intact dsDNA from the HamA active site. (I) Model of threshold activation of Hachiman. (J) Proposed mechanism of Hachiman activation. See also Figure S7.

Our data are consistent with a model in which Hachiman triggers Abi when 3′ ssDNA enters the HamB active site. Although the exact mechanism of DNA damage sensing by HamB remains unclear, we observed formation of ATP-dependent HamA*B-DNA complexes *in vitro* (Figure 3I). We used cryo-EM to visualize this state. HamA*B was incubated with plasmid DNA and ATP for 30 min of reaction, after which the specimen was frozen (Figure 7A). In the resulting micrographs, many particles can be seen binding intact plasmid DNA (Figure 7B). In two-dimensional (2D) class averages of plasmid-bound particles, complete HamA*B complexes are seen, with duplex DNA spanning the protein and bending slightly at the point of contact (Figures 7C and S7A). The angle of the DNA in this “scanning” state is orthogonal to DNA resolved in the “loading state” in HamB-DNA structures (Figure 7D). Masked 3D classification and unbiased alignments produced a map with a 3.2-Å nominal resolution, with lower resolutions (5–7 Å) for the plasmid DNA, although the major and minor grooves in the central region are apparent (Figures 7E, 7F, and S7A–S7E; Table S1). The dsDNA interacts with the RecA2 loop region (Figure 7E). There are few differences between the rest of the complex and the apo HamAB structure (Figure 2B). In the molecular model, duplex DNA occupies the same location as RecA2 loop—we could not find an alternate conformation of the loop structure, leading us to conclude that it becomes disordered once DNA is bound (Figure 7F). ATP occupies the binding pocket, consistent with biochemical results (Figures 3I and 3J). Contacts made with ATP are in agreement with predictions for HamB Walker and helicase motifs (Figures 7G, S3H, and S3I). Our observations suggest that HamAB surveys DNA in an alternative scanning mode that may enable monitoring of dsDNA. In this mode, which is facilitated by the RecA2 DNA loop, DNA is restrained from the nuclease active site (Figure 7H). Upon phage infection, or conditions that cause elevated levels of DNA damage, HamAB activates by loading of a DNA end, or potentially by recognition of specific DNA structures involved in damage responses such as displacement loops (Figure 7I). Entrance of ssDNA into the helicase active site triggers ATPase activity, leading to structural rearrangements enabling HamA activation (Figure 7J).

## DISCUSSION

Our results reveal that the Hachiman prokaryotic defense system is a nuclease-helicase complex, HamAB, which responds to changes in genome integrity. Upon contact with a free ssDNA end, the end inserts into the HamB active site to induce ATP hydrolysis, HamB ratcheting and activation of the HamA nuclease. Activated Hachiman catalyzes DNA degradation, creating phantom cells cleared of both phage and host DNA, reminiscent of NucC-mediated clearing in some type III CRISPR-Cas systems.^53^ That Hachiman separates its nuclease and helicase components between two subunits, HamA and HamB, may be intrinsically linked to its robust Abi phenotype. *Trans* nuclease activity from HamA activation could initiate a positive feedback loop. Elevated DNA damage due to HamA activation may then enable other Hachiman complexes to detect new sites of DNA damage, amplifying the immune response. We propose that major changes in genome integrity, such as host genome degradation or recombination-dependent replication by dsDNA phages,^26^ result in accumulated damage. Extensive damage “tips the scales” toward Hachiman activation, leading to Hachiman-induced DNA damage and phage restriction through Abi. Our results implicate genome integrity as an important battleground during viral infection.

The spread of “selfish” mobile genetic elements significantly alters nucleic acid metabolism in the cell. For instance, phage T4 degrades the host genome to preferentially replicate and suppress antiphage activities.^54,55^ However, viral teleonomy involves tradeoffs: rapid replication leads to higher error rates,^56^ increasing the frequency of lesions and stalled replication forks. This is compounded by damage inflicted on the phage genome by other defense systems.^57^ Phages engage in orthogonal homologous recombination to compensate, a process that universally involves a free 3′ ssDNA end and displacement-loop intermediates.^58,59^ We propose that Hachiman senses and activates in response to motifs associated with stress on the integrity of host or phage DNA,^60^ enabling viral sensing across a range of infection strategies. This explains the broad-spectrum protection conferred by Hachiman. The exact identity of 3′ end-containing DNA structures that activate Hachiman during phage infection is an area of future interest. We imagine that Hachiman is evolutionarily tuned to activate only when DNA damage is too severe for the host DNA repair machinery to remedy, or if invading entities undergo uncontrolled replication.^26,58^

A recent study proposed that phage-encoded SSB activates *B. cereus* Hachiman.^34^ However, our data suggest that this mode of activation is a proxy for the true activator of Hachiman encoded in DNA structure. One parsimonious explanation is that expression of phage SSB is incompatible with host replication and recombination machinery, while the phage preferentially replicates its own genome. This could result in DNA damage or produce structures such as displacement loops that appear as DNA damage to Hachiman. *In vitro* activation of ECOR31 HamAB activity did not require SSB, nor was a differential effect seen when comparing phage and host SSB, consistent with observations in the XPD SF2 helicase.^61^ Considering the minimal sequence and structural homology between the two stimulatory SSBs, we consider it unlikely that either activates HamB by protein-protein interactions. Our results imply that other defense systems believed to be SSB-activated may be stimulated by DNA damage.^36,62^

As Hachiman both senses and induces DNA damage, Hachiman must be regulated during normal cell activities. We find ectopic Hachiman expression to be mildly toxic (Figure S7F), in line with prior work,^34^ as a fitness cost of carrying a potent and general immune system. During normal cell activities, DNA damage is likely limited and transient thanks to proofreading activities of the native DNA repair machinery (Figure 7I).^63^ How Hachiman interfaces with evolutionarily conserved DNA repair pathways to limit premature activation is of future interest. Cells harboring Hachiman wield broad-spectrum defense, but at the risk of autoimmunity.

We identify HamA, previously DUF1837, as the effector nuclease responsible for DNA clearance. Compared with structural homologs in type IIS RM systems, HamA contains insertions that mediate interactions with HamB. Considering the HamA interaction domain (NAH) is present in all HamBs, and that this interaction domain is absent in close relatives in the Ski2 subfamily (Figure S5M),^64,65^ we propose HamA insertions were acquired during Hachiman evolution to enable nuclease regulation and allosteric activation. Another distinguishing feature of HamB is the loop on the crown of the RecA2 domain that both facilitates scanning of intact dsDNA and forms contacts with DNA during loading into the helicase active site. Comparison with predicted structures of HamB orthologs confirms that either the RecA2 loop or a highly positively charged patch exists at this position, suggesting that dsDNA sliding or binding may be a common feature in Hachiman defense (Figure S7G). Other immune helicases have been proposed to “scan” DNA or RNA for pathogenic signatures.^13,66^ Future studies should address the nature of HamAB DNA surveillance with single-molecule techniques.

The AbpAB antiphage defense system was described before the identification of Hachiman.^67^ AbpAB encodes a nuclease (AbpA) and a Ski2-like helicase (AbpB) with combined activity against DNA. The N-terminal domain of AbpA is similar to the Cap4 endonuclease domain from CBASS systems.^68^ The C-terminal domain of AbpA is remarkably genetically and structurally similar to HamA, but contains catalytically inactive residues within the HamA active site (Figures 2, S2A, and S7H). AbpAB was recently shown to activate in response to mitomycin C-induced DNA damage, in agreement with our data.^36^ Based on structural and functional homology, we propose AbpAB is a Hachiman variant with an N-terminal fusion in the HamA homolog AbpA (Figures S2A and S2B). Why AbpAB would encode a catalytically inactive form of the HamA nuclease while carrying an additional, distinct endonuclease remains enigmatic.

Beyond antiphage immunity, Ski2-like helicases variably accept RNA or DNA substrates.^30^ Our biochemical results demonstrate that HamB is more functionally similar to the Ski2-like DNA helicase Hel308 than Ski2 RNA helicases involved in RNA regulatory processes such as splicing and mRNA decay. Hel308 is conserved in archaea and metazoans, but is absent in bacteria and fungi.^69,70^ Like HamB, Hel308 has a wide substrate scope, with a preference for 3′ to 5′ DNA unwinding.^21,65^ Human Hel308 (HELQ) is involved in DNA repair and was shown to localize to sites of DNA damage induced by mutagens *in vivo*.^71–73^ We observed an analogous response to DNA damage induced by drugs in cells harboring Hachiman. Genetic similarities and functional symmetries between HamB and Hel308 suggest an evolutionary relationship.

This study provides structural and biochemical analyses of Hachiman function that extend our understanding of prokaryotic immune mechanisms. The Hachiman sensor helicase HamB has surprising functional similarities to the archaeal and metazoan DNA repair helicase Hel308, which may explain its general activity against DNA damage. Genome integrity sensing may be a more general role of helicases in immune systems beyond Hachiman.

### Limitations of the study

In this study, we propose a mechanism for Hachiman immunity, but several questions remain. We resolved only partial strand unwinding in HamB-DNA structures, though complete melting was observed for short duplexes *in vitro* (Figure 3F). The role of HamB translocation, and whether it contributes to immunity beyond sensing DNA damage and releasing HamA, is unclear. Relatedly, whether HamB actively “passes” HamA to loaded DNA (*cis* cleavage) or simply releases the nuclease for degradation (*trans* cleavage) is an open question. The relative proportion of *cis* to *trans* cleavage is probably salient to the level of defense conferred by Hachiman. Structural views of HamA alone and bound to target DNA could further elucidate the molecular mechanism. Phage-encoded peptide inhibitors of Hachiman were recently reported, though the mechanism of action is mysterious.^74^ Structural data from this work will guide future studies exploring how phages counteract Hachiman defense.

## RESOURCE AVAILABILITY

### Lead contact

Further information and requests for resources and reagents should be directed to and will be fulfilled by the lead contact, Jennifer A. Doudna (doudna@berkeley.edu).

### Materials availability

Plasmids for wild-type Hachiman loci, protein purification, and select mutants of Hachiman generated in this study have been deposited to Addgene (ID: 223362–223372). This study did not generate new unique reagents.

### Data and code availability

Structure coordinates and corresponding density maps have been deposited at the Protein Data Bank (PDB) and Electron Microscopy Database (EMD), respectively, under the following accessions: E. coli ECOR31 apo HamAB, PDB: 8VX9, EMD-43613; E. coli ECOR31 HamB-DNA (conformation 1), PDB: 8VXA, EMD-43615; E. coli ECOR31 HamB-DNA (conformation 2), PDB: 8VXC, EMD-43616; and E. coli ECOR31 HamA(E138A,K140A)B-plasmid DNA, PDB: 8VXY, EMD-43643. Additional raw data have been deposited at Figshare and are publicly available (Figshare project: 217540). This paper does not report original code. Any additional information required to reanalyze the data reported in this paper is available from the lead contact upon request.

## STAR★METHODS

### EXPERIMENTAL MODEL AND STUDY PARTICIPANT DETAILS

#### Bacterial strains and bacteriophages

For standard cultivation, *E. coli* strains listed in the key resources table were grown in LB media at 37 °C at 250rpm. Whenever applicable, media was supplemented with carbenicillin (100 μg mL^−1^), chloramphenicol (20 μg mL^−1^) or kanamycin (50 μg mL^−1^) to ensure plasmid maintenance. For bacterial assays, strains were maintained as 25% (v/v) glycerol stocks at −80°C.

Phage propagation was performed using commonly employed protocols. In general, phages were propagated at 37C in LB Lennox media using an initial MOI of 0.1 and host *E. coli* BW25113 (F- DE(araD-araB)567 lacZ4787(del)::rrnB-3 LAM- rph-1 DE(rhaD-rhaB)568 hsdR514). Phage G17 was propagated on *E. coli* DSM 103255. Phage Goslar was propagated on *E. coli* MC1000. Phages MS2 and M13 were propagated on *E. coli* dh5a F’ cells with added 1 mM CaCl2. All phage titers were determined on their assay hosts harboring a negative control plasmid (pBA635). Infections were carried out as detailed in each section. Bacteriophages used in this study are listed in the key resources table.

### METHOD DETAILS

#### Helicase and Hachiman phylogenetic analysis

Proteins chosen for phylogenetic analysis were from DefenseFinder RefSeq db with a RefSeq Protein ID.^16,17^ Proteins from non-Restriction-Modification (RM) defense systems encoding a SF1/SF2 helicase domain were further selected for phylogenetic comparison: AbiR (AbiRc), Azaca (ZacC), BREX (BrxHI, BrxHII), DISARM (DrmA, DrmD), Dpd (DpdE, DpdF, DpdJ), Druantia (DruE), Gabija (GajB), Gao_RL (RL), Hachiman (HamB), Hhe (HheA), Hna (Hna), Mokosh (MkoA, MkoC), Nhi (Nhi), PsyrTA (PsyrT), Rst Helicase+DUF2290 (Helicase), Shango (SngC), Type I CRISPR-Cas (Cas3), Type IV CRISPR-Cas (Csf4/DinG), and Zorya (ZorD). Helicase proteins without RefSeq protein IDs or not tracked in DefenseFinder (ex. Hma) and from RM defense systems (ex. Type I (Type_II_REases) and Type III RM (Type_III_REases)) were not included in the analysis.

For analysis of SF1 and SF2 helicases, 4 randomly chosen examples of the above defense-associated SF1/SF2 helicases were selected and compared to a curated set of SF1/SF2 helicase core domains.^12^ To focus our analysis on the helicase core domain, we manually curated the helicase core domain of defense-associated helicases through structural alignment followed by sequence alignment. First, a predicted AlphaFold2 structure of each defense-associated helicase was aligned to the core helicase domain of HamB (PDB ID: 8VXA, this work, residues 286–472, 500–731)^17,75,91^ within the core helicase domain were inferred. Next, for each helicase type, representative helicases were aligned to the corresponding annotated reference using MUSCLE (default parameters), core helicase annotation extracted and insertions removed in Geneious Prime v2023.2.1^77,78^To build the helicase tree, sequences were concatenated with a curated set of SF1/SF2 core helicase domains,^12^ aligned using ClustalOmega (default parameters), phylogenized using IQ-TREE (-bb 1000, -m MFP (optimal model: LG+R8)), bootstraps inferred using UFBoot2 and visualized using iTOL.^78–80,92^ Clades were inferred by using bootstrap values ≥ 85 followed by analysis of associated sequences.

For analysis of full-length HamA and HamB, proteins were combined and clustered using mmseqs2 (80% coverage, 80% sequence identity).^81^ HamA nucleases and HamB helicases investigated in this study from ECOR04, ECOR28, and ECOR31 and from prior work^14,36^ were spiked into this collection of non-redundant protein sequences for in-group analysis. Sequences were aligned with ClustalOmega (default parameters), phylogenized using IQ-TREE (-bb 1000, -m MFP (optimal model: LG+F+R8 (HamA), LG+F+R10 (HamB))) and visualized using iTOL.^78,79,92^ Clades were inferred by using bootstrap values ≥ 85 in the HamB followed by analysis of spiked-in in-groups.

#### Identification and visualization of Hachiman-containing loci

Hachiman loci and nearby defense systems were identified in ECOR04 (NZ_QOWP01000021), ECOR28 (NZ_QOXN01000003) and ECOR31 (NZ_QOXQ01000009) using PADLOC.^18^ Gene annotations were visualized and represented using dna_features_viewer.^82^

#### Plasmid and strain construction

All new plasmids in this study were constructed through PCR, gel extraction (Zymo D4001) and through Gibson assembly^93^ or Golden Gate assembly.^94^ DNA PCR templates for wildtype and mutant Hachiman loci originated from isolated gDNA (Qiagen DNeasy Blood & Tissue Kit, 69504) from ECOR04, ECOR28 and ECOR31. For most *E. coli* assays, Hachiman loci were cloned under pTet control into a p15a vector with chloramphenicol (Cm, Sigma) resistance. For *E. coli* assays involving low-copy vectors, Hachiman loci were cloned under pJEX control into a SC101 vector with Kanamycin (Kan, Sigma) resistance. For protein expression and purification, Hachiman loci were cloned under T7 control in a high copy vector with carbenicillin resistance. In general, plasmids were propagated in dh10b genotype *E. coli* (F – mcrA Δ(mrr-hsdRMS-mcrBC) endA1 recA1 ϕ80dlacZΔM15 ΔlacX74 araD139 Δ(ara, leu)7697 galU galK rpsL (StrR) nupG λ-) (Intact Genomics). For subsequent phage assays, some plasmids were transferred to *E. coli* MC1000, *E. coli* ECOR47 or *E. coli* dh5a F’ where indicated. For protein expression and purification, plasmids were transformed into BL21 AI genotype *E. coli* (F- ompT hsdSB (rB-mB-) gal dcm araB::T7RNAPtetA). Plasmids used in this study are listed in Table S2. All plasmids used in this study were sequenced-confirmed by full-plasmid sequencing services using Primordium.

#### Plaque assays

Phage plaque assays were performed using a double agar overlay protocol. Briefly, cultures were grown overnight at 37 °C and 250 rpm. To form overlays, 100 μL of saturated culture was mixed with molten LB Lennox agar (0.7% w/v agar, 60°C). For assays involving G17 or Goslar a less-dense agar concentration was used (0.35% w/v). The agar-bacterial mixture was supplemented with Cm to a final overlay concentration of 34 μg/mL and anhydrotetracycline (aTc) (Sigma) concentration of 20 nM. For phages M13 and MS2 an CaCl_2_ was added to a final concentration of 1 mM. The top agar and bacterial mixture was poured onto a 5 mL LB Agar and Cm plate and left to dry under microbiological flame for 15 minutes. For plaque assays involving low copy Hachiman loci (strains containing pBA1558 or pBA1747), the agar-bacterial mixture was supplemented with Kan to a final overlay concentration of 50 μg/mL and no inducers were added. Phages were diluted 10X in SM buffer (Teknova) and 2 μL of each dilution were spotted onto the top agar and left to dry under microbiological flame. Once dry, plates were incubated at 30°C for 12–16 hours. Plates were scanned in a standard photo scanner and plaque forming units (p.f.u) were enumerated, keeping note of changes in plaque size relative to a negative control. During assays where “lysis from without”^95^ phenotypes were observed, we interpreted these as a lack of productive phage infection and were approximated as 1. p.f.u. at that concentration. Efficiency of plaquing (EOP) calculations were calculated as mean(p.f.u.condition)/ mean(p.f.u.negativecontrol) in Python. The negative control is defined as catalytically deactivated RfxCas13d under pTet control using an RFP-targeting guide.^96^ All plaque assays were performed in biological triplicate. Visualizations were performed using GraphPad Prism or Seaborn in Python.

#### Bacteriophage liquid growth assays and phage production estimation

Liquid phage experiments were performed in a Biotek plate reader using LB + Cm +20 nM aTc media. Strains containing a Hachiman-expressing plasmid (pBA1370) or negative control (pBA1467) were grown overnight at 37 °C and 250 rpm. Strains were seeded into a 96-well microplate reader plate (Corning 3903) at a cfu of ~8e6 cfu per well in 200 μL media. For phage experiments, EdH4 was diluted to maximal concentration of 2e10 PFU/mL in assay media,subsequently diluted and 4μL of phage was added to achieve defined MOIs during infection. Growth was monitored in a Biotek Cytation 5 plate reader for 10 hours at 800 rpm shaking at 37°C with OD600 readings every 5 minutes. To estimate free phage particle production at the end of liquid phage assays, wells from infections at defined MOIs were pelleted and the supernatant collected. Phage titers were enumerated via plaque assay on *E. coli* harboring pBA635 and free phages were determined by dividing by the effective titer at time 0. All liquid phage assays were performed in biological triplicate, sourcing strains from independent overnights. Data were plotted using the matplotlib and seaborn package in Python.

To estimate free phage particle production from a single round of infection, 5 mL cultures were inoculated with ~2e8 cfu of *E. coli* harboring either a Hachiman-expressing plasmid (pBA1370) or negative control (pBA1467) in LB + Cm +20nM aTc media. Cultures were incubated at 37°C, 250 rpm for 15 min. Following incubation, ~5e6 pfu of phage EdH4 was added to each culture to achieve a low MOI of ~0.025. Infections were allowed to proceed at 37°C, 250 rpm. At 0, 30, 60 and 90 minutes post infection, 200μL of infection was sampled, pelleted, supernatant extracted and stored on ice until all samples were collected. Phage titers were enumerated via plaque assay on E. coli harboring pBA635 and free phages were determined by dividing by the effective titer at time 0. Low MOI liquid infection was performed in biological triplicate, sourcing strains from independent overnights. Data were plotted using the matplotlib and seaborn package in Python.

#### Bacterial liquid growth assays

For Hachiman toxicity-profiling experiments, ig10b strains containing a Hachiman- (pBA1370) or a Hachiman mutant- (pBA1464, pBA1465, pBA1467, pBA1469) expressing plasmid were grown overnight at 37°C. Strains were seeded into a 96-well microplate reader plate (Corning 3903) at a cfu of ~8e6 cfu per well in 200μL LB + Cm media. A concentrated stock of aTc was diluted 10X in LB + Cm media and 4 μL added to each well to achieve concentrations of 0, 2, 20 and 200 nM aTc.

For antibiotic sensitivity experiments, an *E. coli* strain lacking transposable elements including prophages (MDS42, key resources table) was employed. Strains containing a Hachiman- (pBA1370) or a Hachiman mutant- (pBA1467, pBA1468 or pBA1469) expressing plasmid or a vector control (pBA1801) were grown overnight at 37°C. Strains were seeded into a 96-well microplate reader plate (Corning 3903) at a cfu of ~8e6 cfu per well in 200μL LB + Cm + 20 nM aTc media. Antibiotics (nalidixic acid (Sigma), novobiocin (Sigma), gentamycin (Sigma), mitomycin C (Sigma) and bleomycin (Sigma)) experiments, antibiotics were diluted 2X in LB + Cm media and 4 mL added to each well to achieve final, maximal concentrations of 30 μg/mL, 1000 μg/mL, 40 μg/mL, 2 μg/mL or 40 μg/mL, respectively. Growth was monitored in a Biotek Cytation 5 plate reader for 16 hours at 800 rpm shaking at 37°C with OD600 readings every 5 minutes. Minimum inhibitory concentrations of antibiotic were determined by investigating the lowest concentration of antibiotic that consistently grew to a lower carrying capacity in the vector control than the untreated condition.

All assays were performed in biological triplicate, sourcing strains from independent overnights. Data were plotted using the seaborn package in Python.

#### DNA Substrate Preparation

Oligonucleotides were synthesized by Integrated DNA Technologies (Coralville, IA). Substrates used in unwinding assays were prepared by mixing the fluorescent or larger strand with a 1.5-fold excess of the non-fluorescent strand in hybridization buffer (20 mM Tris-HCl (pH 7.5), 25 mM KCl, 10 mM MgCl_2_), and heating to 95 °C followed by slow cooling to room temperature for at least an hour. Annealed substrates were purified on an 8% native PAGE gel.

#### Protein expression and purification

All Hachiman purification constructs were N-terminally tagged with 10xHis-MBP-TEV. For complex purification vectors in the native locus format, only HamA was tagged with 10xHis-MBP-TEV. After transformation into BL21-AI *E. coli*, cells were grown to an optical density of ~0.6 then induced overnight at 16°C with 0.5 mM isopropyl-β-D-thiogalactopyranoside (IPTG) and 0.1% L-arabinose. Cells were harvested and resuspended in lysis buffer (20 mM HEPES, pH 8, 500 mM NaCl, 10 mM imidazole, 0.1% Triton X-100, 1 mM Tris (2-carboxyethyl)phosphine (TCEP), Complete EDTA (ethylenediaminetetraacetic acid)-free protease inhibitor (Roche), 0.5 mM phenylmethylsulfonyl fluoride (PMSF) and 10% glycerol). Cells were lysed by sonication, then clarified by centrifugation. The clarified lysate was incubated with Ni-NTA resin for 1 hr. The resin was washed with wash buffer (20 mM HEPES, pH 8, KCl mM NaCl, 10 mM imidazole, 1 mM TCEP, and 5% glycerol), then bound protein was eluted with wash buffer supplemented with 300 mM imidazole. Eluate was then run over an MBPTrap column (GE Healthcare), washed with MBP/SEC wash buffer (20 mM HEPES, pH 8, 150 mM KCl, 1 mM TCEP, and 5% glycerol), and eluted with MBP/SEC buffer supplemented with 10 mM maltose. Eluted protein from the MBPTrap column was treated with TEV protease overnight. Protease-treated samples were concentrated and run on either a Superdex 200 10/300 GL column (Cytiva) for HamA or HamB solo constructs, or a Superose 6 increase 10/300 (Cytiva) for HamAB complex preparations. Aliquots were snap-frozen in liquid nitrogen for later use.

#### Cryo-EM sample preparation and data acquisition

The apo HamAB complex sample was rerun over a Superose 6 increase 10/300 (Cytiva) column in Cryo-EM buffer (20 mM HEPES, pH 8,100 mM KCl, 1 mM TCEP, and 0.5% glycerol). The HamB-DNA complex sample was prepared by combining 15 μM HamB with 20 μM DNA in Cryo-EM buffer supplemented with 1 mM ATP and 2 mM MgCl_2_ and reacting for 30 min at room temperature. Samples were then purified over a Superdex 200 10/300 GL column (Cytiva) in Cryo-EM buffer. The HamA*B-plasmid DNA sample was prepared by combining HamA*B with 1 μg plasmid in cryo-EM buffer supplemented with 1 mM ATP and 2 mM MgCl_2_. The reaction was incubated at 37°C for 5 min, after which the sample was frozen. Samples were frozen in liquid ethane using a FEI Vitrobot Mark IV cooled to 8 °C at 100% humidity on 2/2 200 mesh UltrAuFoil gold grids (Electron Microscopy Sciences) glow discharged at 15 mA for 25 s (PELCO easyGLOW). In all cases, 4 ul of specimen was applied to the grid and immediately blotted for 5 s with a blot force of 8 units.

For apo HamAB and HamA*B-plasmid datasets, micrographs were collected on a Titan Krios G3 equipped with a GATAN K3 Direct Electron Detector in CDS mode and a BIO Quantum energy filter operated at 300 kV and 81,000x nominal magnification in super-resolution mode (0.465 Å/pix). For the HamB-DNA dataset, micrographs were collected on a Talos Arctica equipped with GATAN K3 Direct Electron Detector operated at 200 kV and x36,000 magnification in super-resolution. All cryo-EM data was collected using SerialEM v3.8.7 software.^83^ Images were obtained in a series of exposures generated by the microscope stage and beam shifts. For the HamAB apo and HamA*B-plasmid datasets, movies were acquired in an 11×11 pattern. For the HamB-DNA dataset, movies were acquired in a 7×7 pattern.

#### Cryo-EM data processing

All Cryo-EM data processing was performed in cryoSPARC (v4.2.0 or v4.3.0).^84^ For the HamAB apo specimen, 4,796 movies were collected and 2× binned to a calibrated pixel size of 1.05 Å. 3,314 exposures were accepted after patch motion correction and patch contrast transfer functions (CTF). First, 5,538,550 particles from blob picking were subjected to 2D classification and *ab initio* reconstruction of 3 classes, yielding a density consistent with a complete heterodimeric AB complex. The initial *ab initio* volume was used to create 100 evenly spaced projection-based templates for further template picking. The 3,505,636 particles from template picking were subjected to 4 class *ab initio* reconstruction, which gave a HamAB density of 865,990 particles. Further 2D classification and 2D rebalancing (with 9 superclasses) were used to mitigate orientation bias and remove rod-shaped particles missing multiple HamB domains, leading to a final set of 309,630 particles. Single-class *ab initio* reconstruction followed by non-uniform refinement with on-the-fly defocus and CTF refinement steps gave the final 2.65 Å map,^85^ which was sharpened using DeepEMhancer.^97^

For the HamB-DNA specimen, 9,133 movies were collected and 2× binned to a calibrated pixel size of 1.12 Å. A total of 8,906 exposures were accepted after patch motion correction and patch CTF. Template picker using templates generated from the HamB AF2 prediction gave the best results and were used to isolate 18,254,957 particles at a box size of 256 pix. Reasonable 2D classes were used to train deep picker, which was used to infer 1,125,114 particles at a larger box (512 pix). *Ab initio* reconstruction followed by non-uniform refinement gave a consensus 2.76 Å density with considerable heterogeneity. Then, 3D Variability Analysis (3DVA) with 3 modes using the ‘simple’ output was used to visualize continuous motion.^98^ 3D classification with 5 classes was used to resolve densities representing the maxima of motion resolved in 3DVA. Class 1 of the 3D classification gave HamB-DNA confirmation 1, which was refined (non-uniform refinement with on-the-fly defocus and CTF optimization) to 2.79 Å and sharpened with DeepEMhancer. Class 0 was refined and sharpened in the same manner, giving HamB-DNA conformation 2 at 2.93 Å.

For the dataset containing HamA*B incubated with plasmid DNA, 3724 movies were corrected for beam-induced motion using patch motion correction, then 2× binned to a calibrated pixel size of 1.05 Å. Contrast transfer function parameters were calculated using patch CTF. Initially, 16,398,369 particles were picked using blob picker from all 3724 micrographs. Multiple rounds of reference-free 2D classification were subsequently performed to remove “bad” particles (i.e., particles in 2D classes with fuzzy or uninterpretable features) yielding 87,084 particles with clear protein characteristics. The particles were then submitted for Topaz training, and the resulting Topaz model was used to pick particles from all 3724 micrographs,^99^ giving a total of 1,322,669 particles. Multiple rounds of reference-free 2D classification were subsequently performed to remove junk particles. After selecting the best classes, 317,540 particles were used for *ab initio* reconstruction of 3 classes. Of the 3 classes, 2 classes were selected for subsequent heterogeneous refinement. Heterogeneous refinement yielded a good class with 205,538 particles, and non-uniform refinement was performed with the particles from this class, yielding a reconstruction at 2.86 Å resolution. Afterward, multiple rounds of reference-free 2D classification were performed again to select for good particles which presented resolvable features from 2D classification, resulting in 103,451 particles selected. Then, *ab initio* reconstruction was performed on the selected particles, and subsequently non-uniform refinement, which resulted in a 2.96 Å reconstruction. Then, a focused 3D classification with 4 classes was performed on the predicted DNA binding region of HamA*B, based on views seen in 2D classification, to classify for DNA-bound HamA*B. To generate the focus mask, an atomic model of B-form DNA was built at the predicted DNA binding region, and then a mask of the predicted DNA binding region was artificially simulated using ChimeraX’s molmap function with subsequent binarization and softening. The solvent mask was generated to contain both the protein and predicted DNA densities. The best class containing 29,904 particles yielded a classification that was enriched for DNA-bound HamA*B. Then non-uniform refinement was performed on those particles, which resulted in a 3.2 Å reconstruction, which was then sharpened with deepEMhancer. For visualization, a composite map of protein regions from the 3.2 Å deepEMhancer-sharpened map and DNA regions from the 3.2 Å sharp map with B-factor adjustment was made by the color zone segmentation function in ChimeraX.

#### Model building

The initial model of HamAB was obtained with the ColabFold.^75^ To build the model, we fit the Colabfold prediction into the experimental HamAB apo density with the fitmap tool in UCSF ChimeraX v1.6.1.^86^ There were significant differences in nearly every region of the structure which required iterative manual refinement with a combination of Coot v0.9.4.1,^87^ ISOLDE v1.6.0,^88^ and Phenix 1.20.1–4487.^89^ The HamAB apo structure served as the initial model for all other models. The HamB-DNA and HamA*B-plasmid DNA models were built in the manner described above. DNA was built *de novo*. In the HamA*B-plasmid DNA dataset, the DNA sequence could not be determined, so DNA was modeled as a 31-mer of A-T to maintain base pair interactions during model building. All models were subjected to a final round of Phenix real-space refinement.

#### NTPase assays

Orthophosphate liberation was determined with a Malachite Green Phosphate Assay kit (BioAssay Systems, Hayward, CA, USA) according to the manufacturer protocol. Briefly, HamB reactions were run in Isothermal Amplification Buffer (henceforth IAB, New England Biolabs, 20 mM Tris-HCl, 10 mM (NH_4_)_2_SO_4_, 50 mM KCl, 2 mM MgSO_4_, and 0.1% Tween^®^ 20 (pH 8.8 at 25 °C). HamB was diluted to 40 nM, and nucleic acid substrates listed in Table S3 were diluted to 100 nM, or 4 ng/μl for plasmid reactions, in a total reaction volume of 80 ul in a clear bottom, flat, black 96-well assay plates (Corning Costar). Reactions were allowed to sit for at least 15 min at ambient temperature before initiation with addition of ATP to 1 mM and incubation at 37°C. Reactions were quenched after 30 min with the addition of activated malachite green reagent. The absorbance values of wells were measured after 20 min of color development at ambient temperature with a Biotek plate reader at 620 nm. Orthophosphate liberation was interpolated against a standard curve with known concentrations of free phosphate. Oligonucleotide substrates are modified from Domgaard et al.^33^

#### HamAB activity assays

Plasmid interference assays were conducted in IAB. Plasmids were diluted to 4 ng/μl, while other dsDNA and ssDNA substrates were diluted as indicated. Nicked and cut plasmid were generated by treatment with Nt.BspQI (New England Biolabs) and BamHI-HF (New England Biolabs), respectively. After DNA addition, ATP was added to a final concentration of 1 mM where indicated. In cases where *E. coli* (QIAGEN) or phage T4 SSB (gp32, New England Biolabs) were diluted directly from concentrated stock to a final concentration of 400 nM, then the SSB-DNA mixture was allowed to rest for 15 min on ice. Reactions were started with addition of MBP-HamA, HamB, HamAB, or HamA*B to a final concentration of 500 nM, unless otherwise noted, and were incubated at 37°C. Reactions were quenched with addition of EDTA to 10 mM at various time points and were imaged on 0.75% TBE agarose gels in the case of plasmids, or 2% agarose gels for ssDNA and short dsDNA experiments. Gels were stained with SYBR-safe and imaged on a ChemiDoc MP (BioRad).

Complex disassembly size exclusion chromatography experiments were run with elevated concentrations of HamAB (10 μM), ATP or AMPPNP (2 mM) and ssDNA (20 μM) in IAB for a total reaction volume of 100 μl. Buffer was supplemented with KCl for a final concentration of 500 mM. Reactions were incubated at 37 °C for 60 min and were then loaded on a Superose 6 increase 10/300 (Cytiva) run with modified SEC buffer (20 mM HEPES, pH 8, 500 mM KCl, 1 mM TCEP, and 5% glycerol).

#### Gel-shift helicase unwinding assays and quantification

Unwinding reactions were carried out at 30 °C in IAB buffer. 100nM of HamB was incubated with 20 nM DNA substrate for 5 min (substrates listed in Table S4), and the reactions with protein were initiated by addition of ATP or AMPPNP to a final concentration of 1 mM. At either 1 min, 5 min, or 20 min, reactions were quenched on ice with STOP Buffer (0.4 U proteinase K (New England Biolabs), 18 mM EDTA, 0.36% SDS, and 9% glycerol. Boiled substrates were incubated at 95 °C for 5 minutes before immediate loading. Samples were electrophoresed until separation in an 8% TBE polyacrylamide gel at 4 °C. Fluorescent bands were imaged using a Typhoon FLA scanner and quantified using Fiji.^87^ The fraction of unwound substrate by HamB was estimated by dividing the intensity of the unwound strand over the sum of the intensities of the unreacted duplex and unwound strand, minus the fraction of unwound substrate from spontaneous unwinding without HamB at 20 minutes, then normalized to the fraction of unreacted duplex without HamB at 20 minutes. The normalized fraction unwound by HamB at time t is given below, where δ is the fraction of ${\text{I}}_{\text{ssDNA},20}$ unwound spontaneously without HamB:

$$t=\frac{\frac{I_{\mathrm{ssDNA},t}}{I_{\mathrm{ssDNA},t}+I_{\mathrm{ssDNA},t}}-\delta }{1-\delta }$$

#### Live single-cell time-lapse and static time-course fluorescence microscopy

Microscopy experiments were performed in biological triplicate. Host cells were grown to OD_600_ 0.3 in LB (+chloramphenicol 30 μg/mL) at 30 °C. 12 μL were spotted and spread on the surface of 1% agarose, 25% LB imaging pads containing 30 μg/mL chloramphenicol and 0.05 nM aTc on single-well concavity glass slides, then incubated for 2–2.5 hours at 30°C without coverslips in a humidor. At this stage, 5 μL of ~2 × 10^10^ PFU/mL EdH4 lysate was spotted and spread onto the imaging pads and the pads were incubated at 30°C without coverslips in a humidor until the desired infection time point. MOI 2 is estimated based on these initial inocula and infection dose after ~2 hrs of incubation before infection and a 30 minute bacterial generation time.

All live cell microscopy was performed on a DeltaVision Elite Deconvolution microscope (Applied Precision, Issaquah, WA, USA). For time-course fluorescence microscopy, imaging pads were stained with 8 μL of dye mix (25 μg/mL DAPI, 3.75 μg/mL FM4–64) and a glass coverslip was placed on top of the pad immediately before imaging at room temperature. For each image, 8 slices in the Z-axis at 0.2 μm increments were collected in each imaging channel (DAPI, FM4–64, brightfield). Exposure times: DAPI = 15 ms, FM4–64 = 150 or 300 ms, brightfield = 80 ms. For time-lapse microscopy, unstained cells were imaged at 5 minute intervals from 5 to 125 mpi at 30°C within the environmental control unit enclosing the microscope stage. 8 slices in the Z-axis at 0.2 μm increments were collected only in the brightfield channel (exposure = 8 ms). Dunnett’s test was performed after repeated-measures one-way ANOVA comparing strains expressing Hachiman (active or inactive) to the control strain at each time point.

Images were deconvolved in DeltaVision SoftWoRx (version 6.5.2). Image analysis was performed using raw images in FIJI (version 2.3.0/1.53q) and GraphPad Prism (version 10.0.0). Figure panels were created in Adobe Photoshop (21.2.0), GraphPad Prism (version 10.0.0), and Adobe Illustrator (24.2). The following are ‘n’ values are the total DAPI-stained DNA cross-sectional area measurements for each condition during time-course fluorescence microscopy (with between 46 and 206 measurements per condition per replicate depending on cell density in individual microscopy fields). - control: Uninfected = 525, 10 mpi = 258, 30 mpi = 254, 50 mpi = 249; HamAB: Uninfected = 261, 10 mpi = 229, 30 mpi = 274, 50 mpi = 240; HamAB*: Uninfected = 264, 10 mpi = 272, 30 mpi = 252, 50 mpi = 285; HamA*B: Uninfected = 230, 10 mpi = 255, 30 mpi = 356, 50 mpi = 277. For time-lapse microscopy, the total number of cells lysed across all three replicates = 121, with 37–44 cells per replicate.

### QUANTIFICATION AND STATISTICAL ANALYSIS

Statistical details for each experiment are found in the figure legend and the accompanying method details. Unless otherwise stated, bar graphs represent the mean of independent biological replicates.

## Supplementary Material

Table S3

Table S4

Table S1

Table S2

1

SUPPLEMENTAL INFORMATION

Supplemental information can be found online at https://doi.org/10.1016/j.cell.2024.09.020.

## Figures and Tables

**KEY RESOURCES TABLE:** 

REAGENT or RESOURCE	SOURCE	IDENTIFIER

Bacterial and virus strains		

*E.coli* dh10b	Intact Genomics	Cat#1284-24
*E.coli* BL21-AI	Fisher Scientific	Cat#C607003
*E.coli* BW25113	Coli Genetic Stock Center	CGSC#7636
*E.coli* dh5a F’	New England Biolabs	Cat#C2992
*E.coli* DSM103255	Deutsche Sammlung von Mikroorganismen	DSM103255
*E.coli* MC1000	Coli Genetic Stock Center	CGSC#6647
*E.coli* ECOR47	V. Mutalik (Patel et al.^23^)	1432555081
*E.coli* ECOR04	V. Mutalik (Patel et al.^23^)	1205536237
*E.coli* ECOR28	V. Mutalik (Patel et al.^23^)	1432650029
*E.coli* ECOR31	V. Mutalik (Patel et al.^23^)	1205377838
*E. coli* MDS42	V. Mutalik (Umenhoffer et al.^41^	GCA_000350185.1
Phage EdH4	DSMZ	MK327930
Phage G17	DSMZ	MK327931
Phage Goslar	J. Pogliano	NC_048170
Phage M13	ATCC	NC_003287
Phage MM02	DSMZ	MK373784
Phage MS2	V. Mutalik	NC_001417
Phage N4	V. Mutalik	NC_008720
Phage PTXU04	DSMZ	NC_048193
Phage SUSP1	S. Adhya	NC_028808
Phage T4	V. Mutalik	NC_000866
Phage T5	V. Mutalik	NC_005859
Phage T7	V. Mutalik	NC_001604

Chemicals, peptides, and recombinant proteins		

SM Buffer	Teknova	Cat#S0249
Chloramphenicol	Sigma	Cat#Cu378
Kanamycin sulfate	Sigma	Cat#60615
Carbenicillin	Goldbio	Cat#C-103-100
Ampicillin	Fisher Scientific	Cat#BP1760-25
Anhydrotetracycline hydrochloride	Sigma	Cat#37919
Nalidixic acid	Sigma	Cat#N8878
Novobiocin	Sigma	Cat#N1628
Bleomycin	Sigma	Cat#B8416
Mitomycin C (MMC)	Sigma	Cat#M4387
4-Nitroquinoline N-Oxide	Sigma	Cat#N8141
Gentamycin	Sigma	Cat#345814-M
isopropyl b-D-thiogalactoside (IPTG)	Goldbio	Cat#I-902
L-(+)-arabinose	Research Products International	Cat#A51000
cOmplete EDTA (ethylenediaminetetraacetic acid)-free protease inhibitor	Roche	Cat#11697498001
MBPTrap HP column	GE Healthcare	Cat#28918780
Superdex 200 10/300 GL column	Cytiva	Cat#28990944
Superose 6 increase 10/300 GL column	Cytiva	Cat#29091598
Isothermal Amplification Buffer	New England Biolabs	Cat#B0537S
*E. coli* single-stranded DNA binding protein (SSB)	QIAGEN	Cat#Y9030L
Phage T4 single-stranded DNA binding protein (SSB)	New England Biolabs	Cat#M0300S
Adenylyl-imidodiphosphate (AMPPNP)	Roche	Cat#10102547001
BamHI-HF	New England Biolabs	Cat# R3136T
Nt.BspQI	New England Biolabs	Cat#R0644S
Proteinase K	New England Biolabs	Cat#P8107S
FM4-64	Fisher Scientific	Cat#T13320
4’,6-diamidino-2-phenylindole, dihydrochloride (DAPI)	Fisher Scientific	Cat#D1306

Critical commercial assays		

DNeasy Blood & Tissue Kit	Qiagen	Cat#69504
Malachite Green Phosphate Assay Kit	BioAssay Systems	Cat#POMG-25H

Deposited data		

E. coli ECOR31 apo HamAB	This paper	PDB 8VX9; EMD-43613
E. coli ECOR31 HamB-DNA (conformation 1)	This paper	PDB 8VXA; EMD-43615
E. coli ECOR31 HamB-DNA (conformation 2)	This paper	PDB 8VXC; EMD-43616
E. coli ECOR31 HamA (E138A,K140A)B-plasmid DNA	This paper	PDB 8VXY; EMD-43643

Oligonucleotides		

Oligonucleotides for ATPase assays, see Table S3.	N/A	N/A
Oligonucleotides for unwinding and nuclease activity assays, see Table S4.	N/A	N/A

Recombinant DNA		

Plasmids for phage defense assays and protein purifications, see Table S2.	N/A	N/A

Software and algorithms		

DefenseFinder v1.2.2	Tesson et al.^17^	https://defensefinder.mdmlab.fr/
ColabFold v1.4.0	Mirdita et al.^75^	https://colab.research.google.com/github/sokrypton/ColabFold/blob/main/AlphaFold2.ipynb
MUSCLE v5	Edgar et al.^76^	N/A
Geneious Prime v2023.2.1	Kearse et al.^77^	https://www.geneious.com/
ClustalOmega v1.2.4	Sievers et al.^78^	http://www.clustal.org/omega/
IQ-TREE v2.3.4	Nguyen et al.^79^	http://www.iqtree.org/
UFBoot2 (in IQ-TREE)	Hoang et al.^80^	N/A
MMseqs2 release 15-6f452	Steinegger and Söding^81^	https://github.com/soedinglab/MMseqs2
dna_features_viewer v3.1.3	Zulkower and Rosser^82^	https://github.com/Edinburgh-Genome-Foundry/DnaFeaturesViewer
PADLOC v2.0.0	Payne et al.^18^	https://github.com/padlocbio/padloc
GraphPad Prism v10.0	GraphPad Software	https://www.graphpad.com/
SerialEM v3.8.7	Matronarde^83^	https://bio3d.colorado.edu/SerialEM/
cryoSPARC v4.2.0, v4.3.0	Punjani et al.^84^	https://cryosparc.com/
DeepEMhancer	Sanchez-Garcia et al.^85^	https://github.com/rsanchezgarc/deepEMhancer
UCSF ChimeraX v1.6	Goddard et al.^86^	https://www.rbvi.ucsf.edu/chimerax/
Coot v0.9.8.93	Emsley et al.^87^	https://www2.mrc-lmb.cam.ac.uk/personal/pemsley/coot/
ISOLDE v1.6	Croll^88^	https://tristanic.github.io/isolde/index.html
Phenix v1.19.2-4158	Afonine et al.^89^	https://phenix-online.org/download/
DeltaVision SoftWoRx v6.5.2	Cytiva	https://download.cytivalifesciences.com/cellanalysis/download_data/softWoRx/7.0.0/SoftWoRx.htm
FIJI v2.3.0/1.53q	Schindelin et al.^90^	https://imagej.net/downloads
Adobe Photoshop v12.2.0	Adobe	https://www.adobe.com/
Adobe Illustrator v24.2	Adobe	https://www.adobe.com/

## Highlights

- Hachiman inhibits phage replication by indiscriminate DNA degradation
- Cryo-EM structures reveal that HamA is a DNase regulated by complexation with HamB
- HamB is a DNA helicase related to archaeal and eukaryotic DNA-repair helicases
- Activation in the absence of phage suggests that Hachiman senses genome integrity
