# Supplementary material for: Genome integrity sensing by the broad-spectrum Hachiman antiphage defense complex
Source: Cell. Author manuscript; Available in PMC 2025 Jul 21. (PMC12278908; doi:10.1016/j.cell.2024.09.020)
Supplement: 1 [file NIHMS2091420-supplement-1.pdf]

Supplemental figures

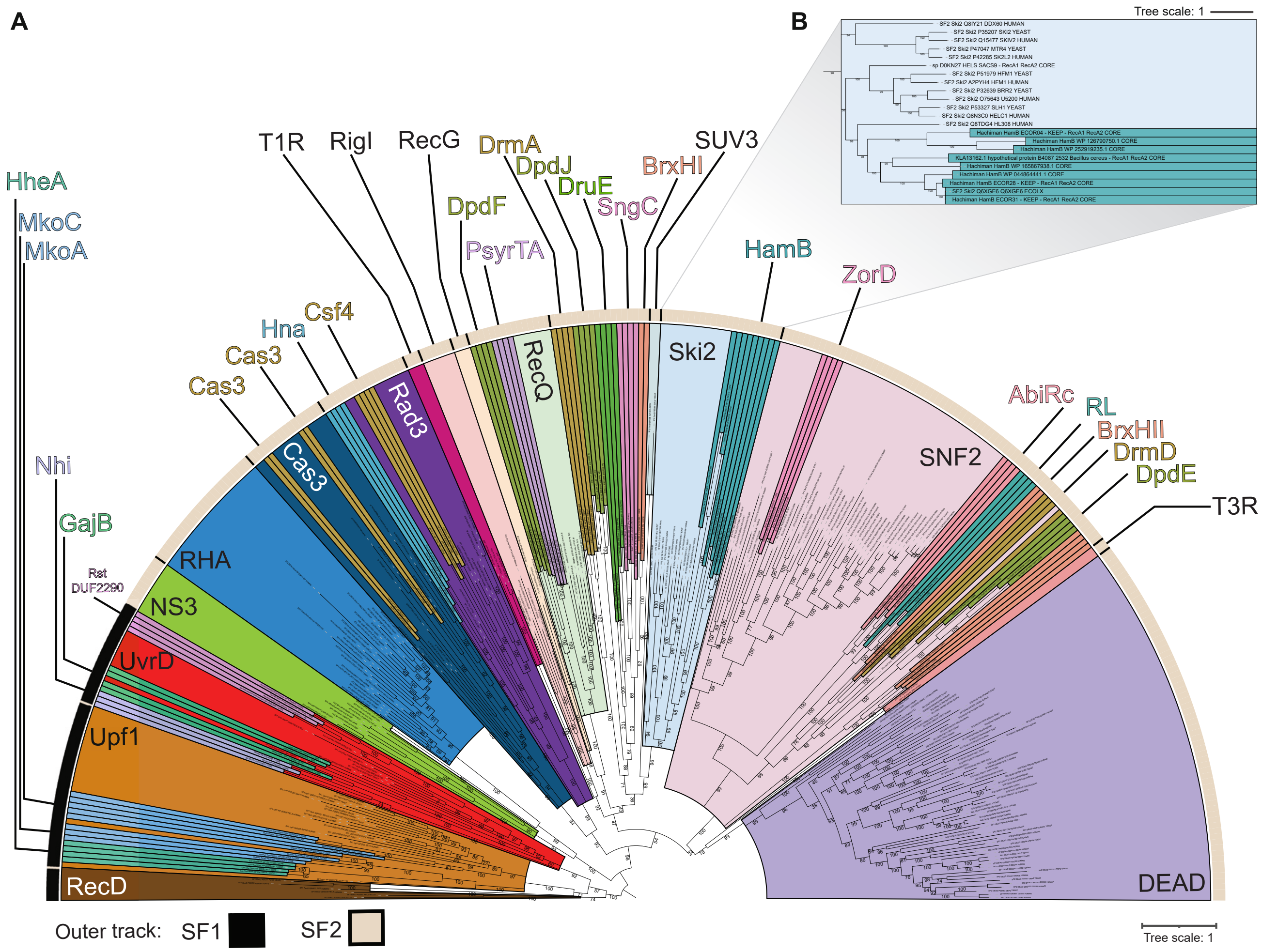

Figure S1. Helicases and Hachiman phylogenetic analysis, related to Figure 1

(A) Annotated phylogenetic tree of phage-defense-system-associated helicase core domains and reference helicases shown in Figure 1B. Bootstrapping values determined by UFBoot2[80] are shown.

(B) Zoomed-in view focusing on represented Ski2 helicases.

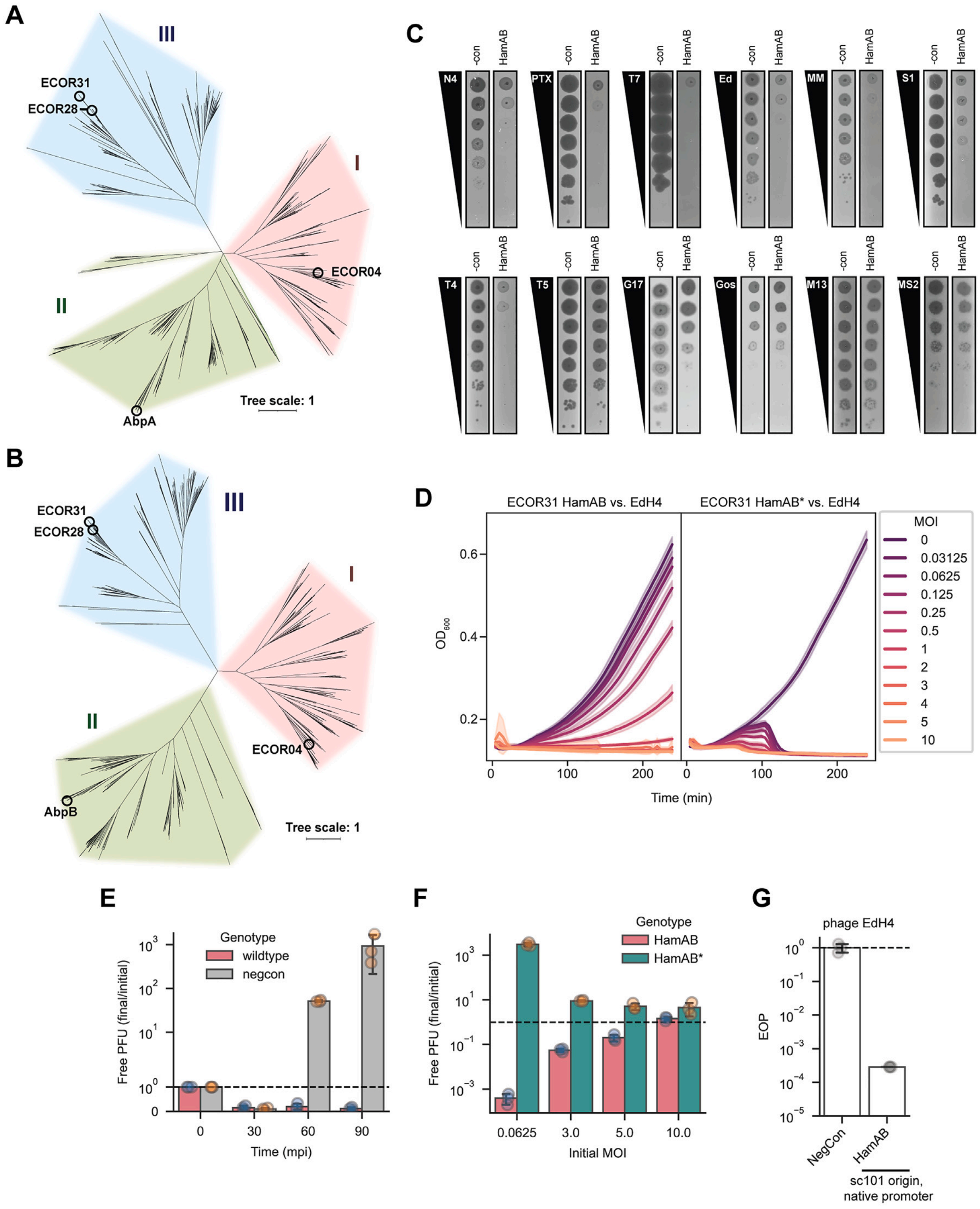

Figure S2. Function and phylogeny of tested HamAB proteins, related to Figure 1

(A and B) Phylogenetic tree of (A) HamA and (B) HamB from DefenseFinder[17] with HamA and HamB sequences from this manuscript and AbpAB[36] are labeled. We assign three potential clades of HamB and their corresponding HamA clades as I–III.

(C) Representative plaque assays for ECOR31 HamAB and phages tested in this study.

(D) Growth curves of *E. coli* expressing ECOR31 Hachiman (left) or HamB mutant (right) during EdH4 infection at specified MOI. Data are shown as mean ± standard deviation across three independent biological replicates.

(E) Phage production assay for EdH4 infection of *E. coli* expressing wild-type Hachiman (red) or HamB mutant Hachiman (green). EdH4 titers were estimated over time by sampling endpoint supernatants of infections from (D). Free plaque-forming unit (PFU) was calculated by dividing the infection titer by the phage titer at 0 min post infection (mpi). Data are shown as mean ± std across three independent biological replicates with individual data points shown.

(legend continued on next page)

CellPress
OPEN ACCESS

(F) Phage replication assay for EdH4 infection of *E. coli* expressing wild-type Hachiman (red) or HamB mutant Hachiman (gray). EdH4 titers were estimated over time by sampling from the supernatant of MOI 0.02 infections. Free PFU was calculated by dividing the infection titer by the phage titer at 0 min post infection (mpi). Data are shown as mean ± std across three independent biological replicates with individual data points shown.
(G) Efficiency of plaquing for EdH4 infection of ECOR31 Hachiman expressed from a low-copy (sc101) plasmid under its native promoter. Data are shown as mean ± std across three independent biological replicates with individual data points shown.

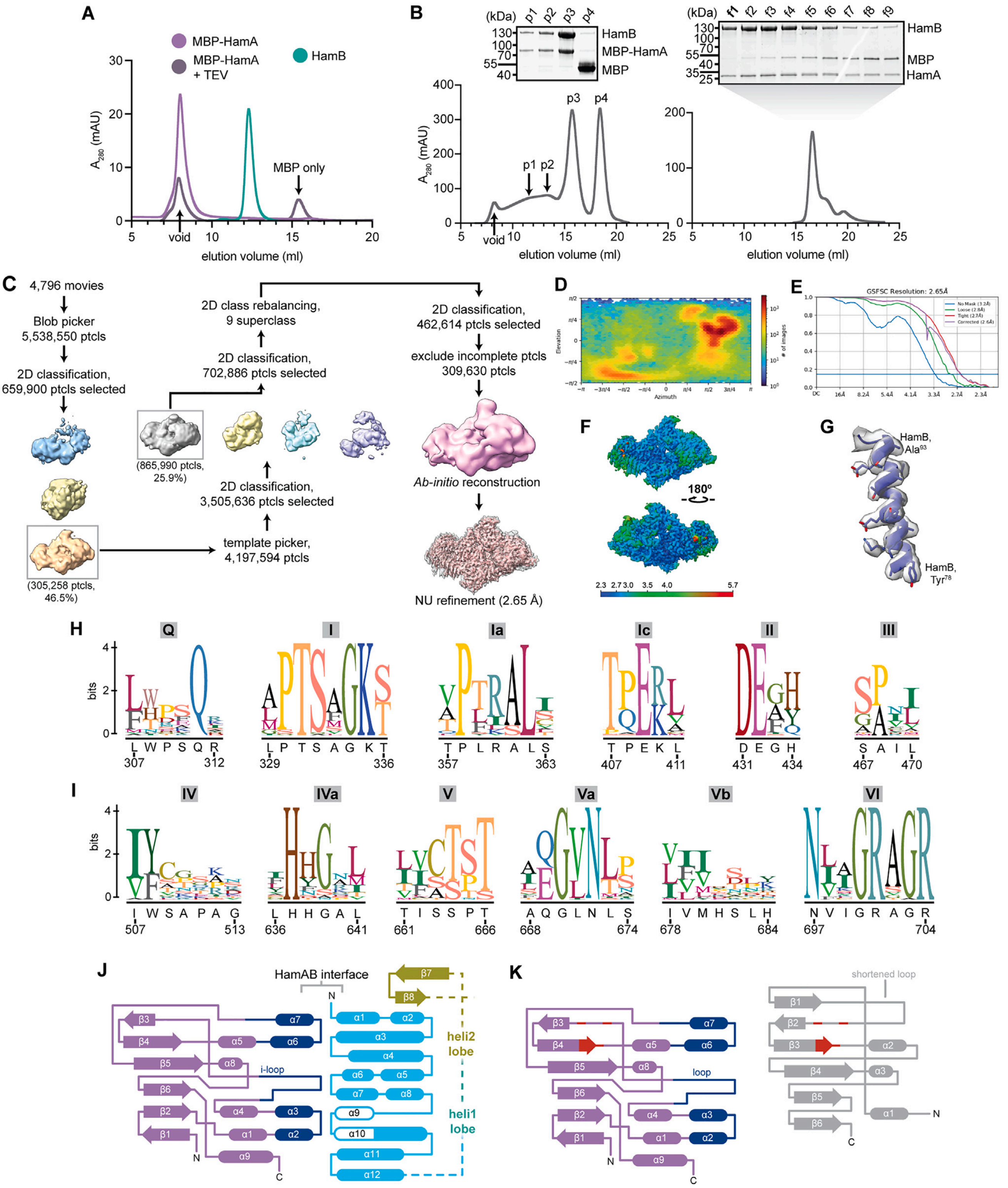

Figure S3. Cryo-EM structure of the HamAB apo complex, related to Figure 2

(A) Size exclusion chromatography traces of MBP-HamA pre-TEV treatment, post-TEV treatment, and HamB alone.

(B) Left, size exclusion chromatography trace of MBP-HamAB and corresponding peaks run on a Coomassie PAGE gel. Right, size exclusion chromatography trace of HamAB after TEV protease treatment, with elution fractions run on Coomassie PAGE gel shown above.

(legend continued on next page)

(C) Particle picking, classification, and refinement strategy to generate the final apo HamAB density.
(D) Orientation distribution of the final particle set.
(E) Gold-standard FSC curve.
(F) Sharpened map colored by local resolution.
(G) Example model-to-map fit.
(H and I) Sequence logos of helicase motifs in the RecA1 (H) and RecA2 (I) domains calculated from the HamB MSA. The residue number and identity of the corresponding sequence in ECOR31 HamB is shown below start and end motif positions.
(J) Secondary structure diagram depicting the HamAB interaction interface.
(K) Comparison of secondary structures of HamA and the *P. aquatile* type IIS restriction endonuclease.

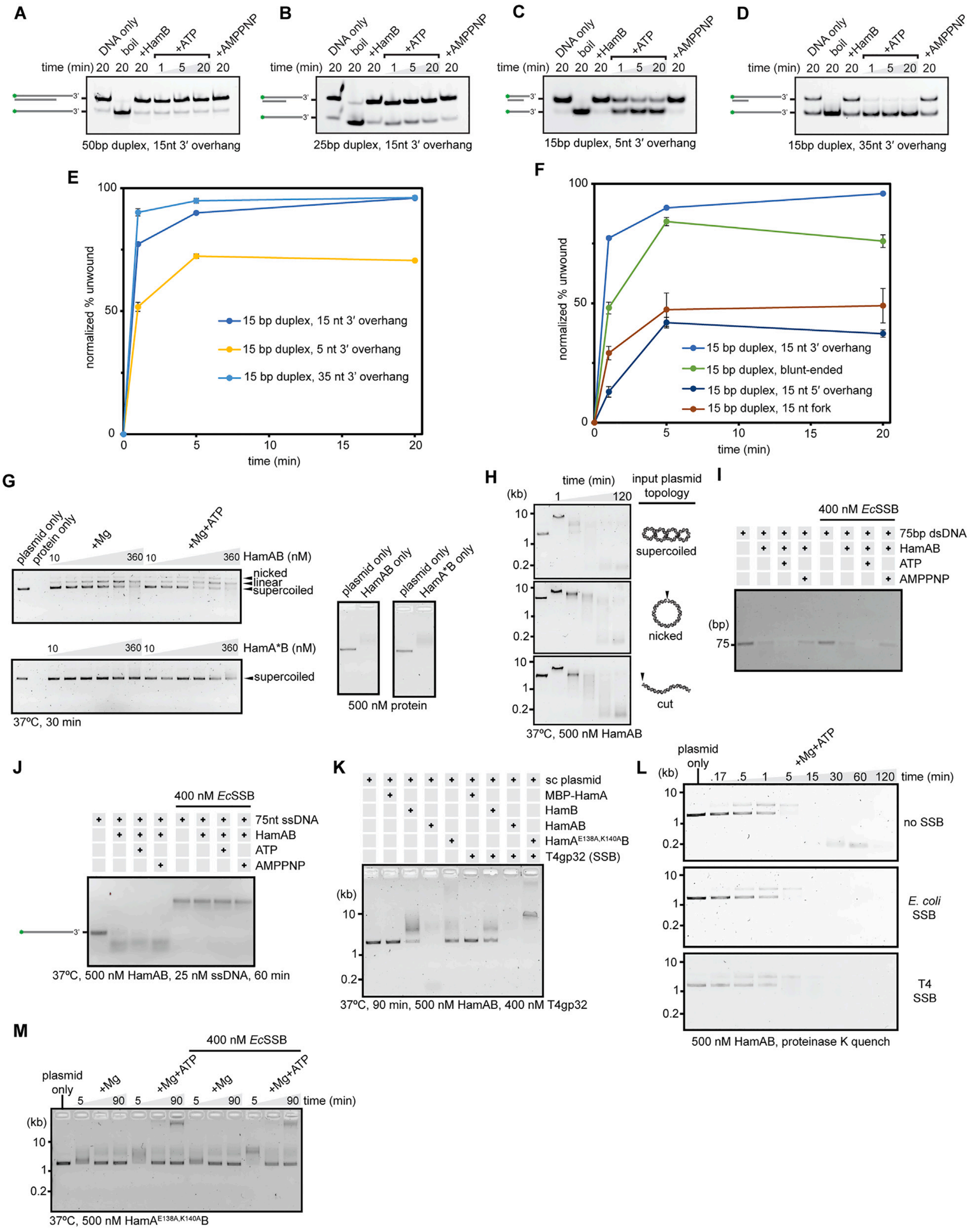

(legend on next page)

CellPress
OPEN ACCESS

Figure S4. Analysis of HamAB biochemical activities *in vitro*, related to Figure 3

(A–D) HamB DNA unwinding assays on substrates with a 50-bp duplex and a 15-nt 3′ OH (A), 15-bp duplex, and 15-nt 3′ OH (B), 15-nt duplex with a 5-nt 3′ OH (D), and 15-nt duplex with a 35-nt 3′ OH (E). DNA substrates are labeled with 5′ FAM. Gels are representative of three independent biological replicates.

(E and F) Normalized percent unwinding of DNA substrates with varying OH lengths (E) and different OHs (F). All substrates are labeled with 5′ FAM. Shown are the mean and standard error of quantifications of three replications of unwinding assays in the format of (A)–(D) normalized against basal unwinding (see STAR Methods).

(G) Left, titration of HamAB WT and HamA*B complexes with plasmid DNA. Right, controls of 40 ng plasmid and 500 nM HamAB and HamA*B, demonstrating small amounts of contaminating DNA in protein preparations.

(H) Time courses of HamAB degradation of supercoiled, nicked, and cut plasmid.

(I) HamAB degradation of a 75-bp dsDNA PCR product, with or without *Ec*SSB.

(J) HamAB degradation of a 5′ FAM-labeled 75-nt ssDNA, with or without *Ec*SSB.

(K) Comparison of Hachiman protein and complex plasmid activities, with or without phage T4gp32 (T4SSB).

(L) Time course comparison of Hachiman-mediated plasmid clearance with or without *Ec*SSB or T4SSB.

(M) Time course of plasmid degradation by HamA*B, with or without ATP and l *Ec*SSB. Conditions in absence of *E. coli* SSB are also shown in Figure 3H.

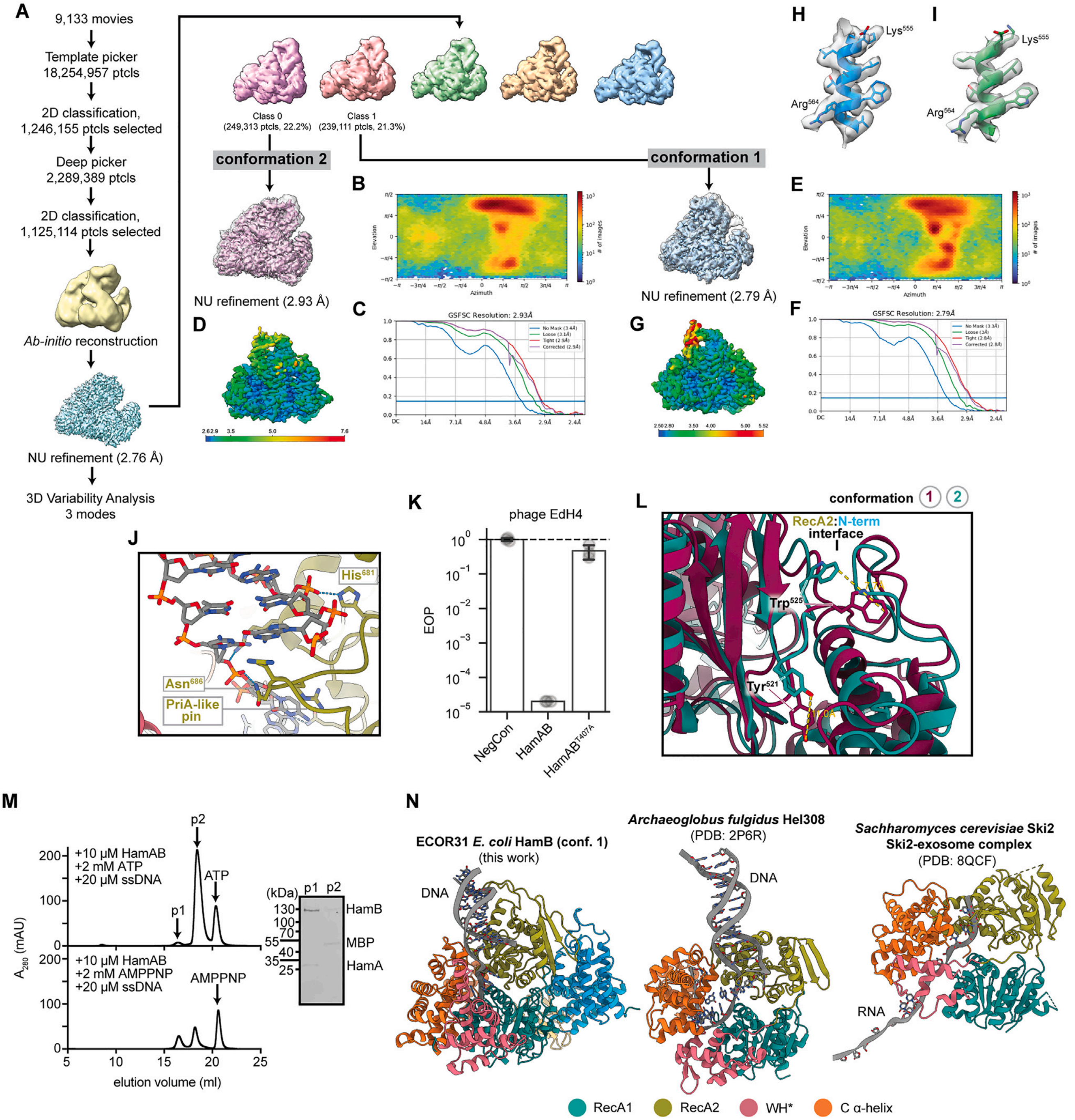

Figure S5. Cryo-EM structure of two HamB-DNA complex conformations, related to Figure 4

(A) Particle picking, classification, and refinement strategy to generate the final HamB-DNA densities for conformations 1 and 2.
(B) Orientation distribution of the final conformation 2 particle set.
(C) Gold-standard FSC curve for conformation 2.
(D) Conformation 2 Sharpened map colored by local resolution.
(E) Orientation distribution of the final conformation 1 particle set.
(F) Gold-standard FSC curve for conformation 1.
(G) Conformation 1 sharpened map colored by local resolution.
(H) Example model-to-map fit for conformation 2.
(I) Example model-to-map fit for conformation 1.
(J) Molecular detail of the PriA-like strand unwinding pin in HamB-DNA conformation 1.

(legend continued on next page)

CellPress
OPEN ACCESS

(K) Efficiency of plaquing for a Hachiman mutant (T407A) deficient in the ability to form a hydrogen bond with the 3′ hydroxyl of ssDNA. Data are shown as mean ± std across three independent biological replicates with individual data points shown.

(L) Detail of the HamB RecA2-NAH interface and comparison of conformational changes.

(M) SEC traces showing ATP-dependent disassembly of HamAB upon addition of ssDNA. Corresponding Coomassie PAGE gel is shown to the right. 10 μM of HamAB are added in both experiments. ssDNA co-elutes with HamB and MBP. Experiments are run in 500 mM KCl.

(N) Comparison of HamB-DNA with other related helicases bound to their substrates. Middle, *A. flugidus* Hel308 (PDB: 2P6R). Right, Ski2:RNA from a structure of the Ski2-exosome complex (PDB: 8QCF). The exosome complex was hidden for clarity.

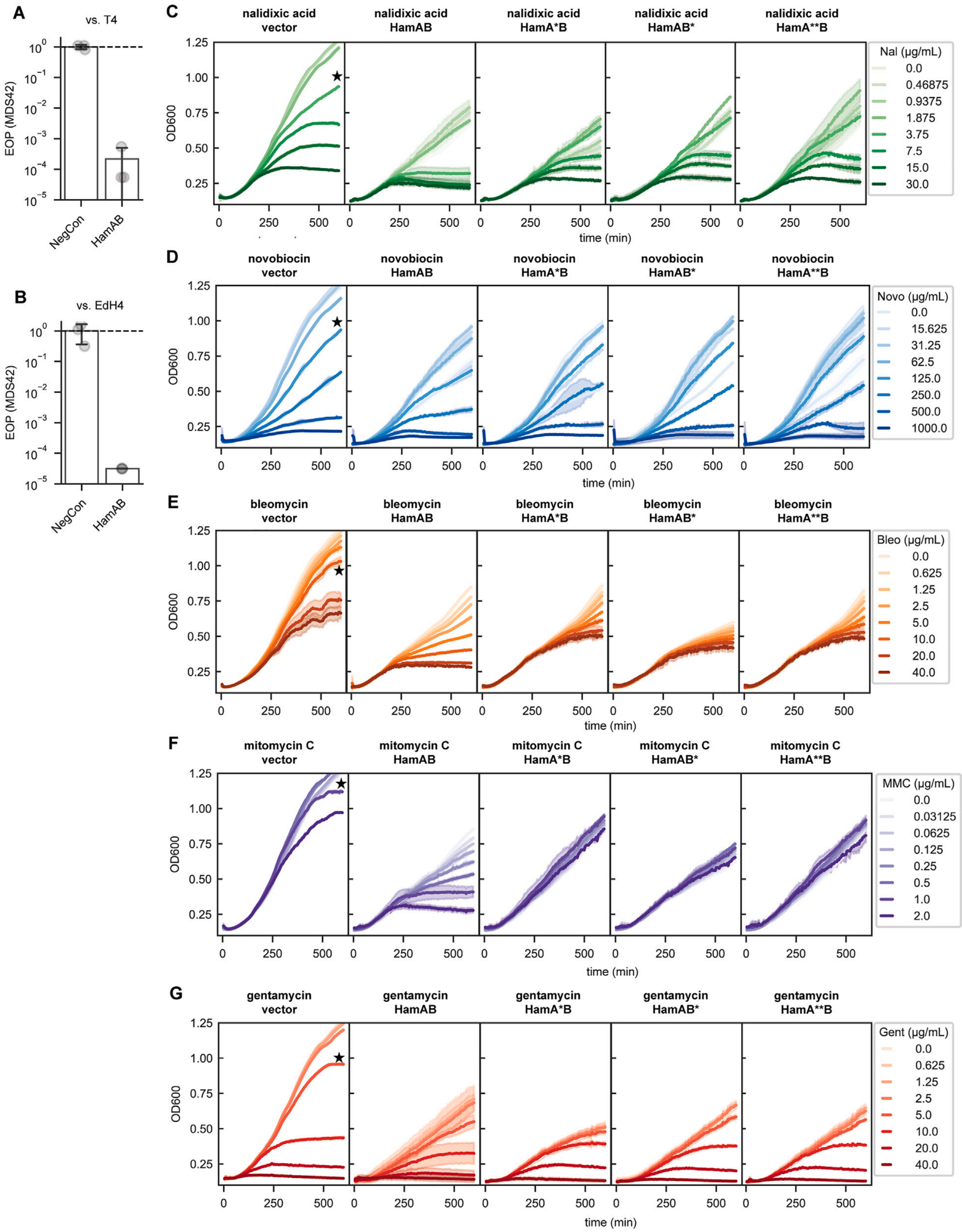

(legend on next page)

Figure S6. Drug-induced DNA damage activates Hachiman, related to Figure 6

(A and B) Efficiency of plaquing for *E. coli* MDS42 expressing wild-type HamAB or a negative control (dCas13d) against phages T4 (A) and EdH4 (B). Data are shown as mean ± std across three independent biological replicates, with individual data points shown.

(C–G) Cell growth of *E. coli* MDS42 expressing a vector control (left), wild-type HamAB (second from left), nuclease-deficient HamA*B (middle), helicase-deficient HamAB* (second from right), and HamA^{D119A}B (HamA**B) (right) at 20 nM aTc in the presence of variable concentrations of nalidixic acid (C), novobiocin (D), bleomycin (E), mitomycin C (F), and gentamycin (G). Growth curves are colored according to condition. The minimum inhibitory concentration without the confounding effects of gene expression determined by a vector control used in Figure 6 is denoted with a star. All growth curves performed in biological triplicate. The mean and standard deviation of each condition are plotted.

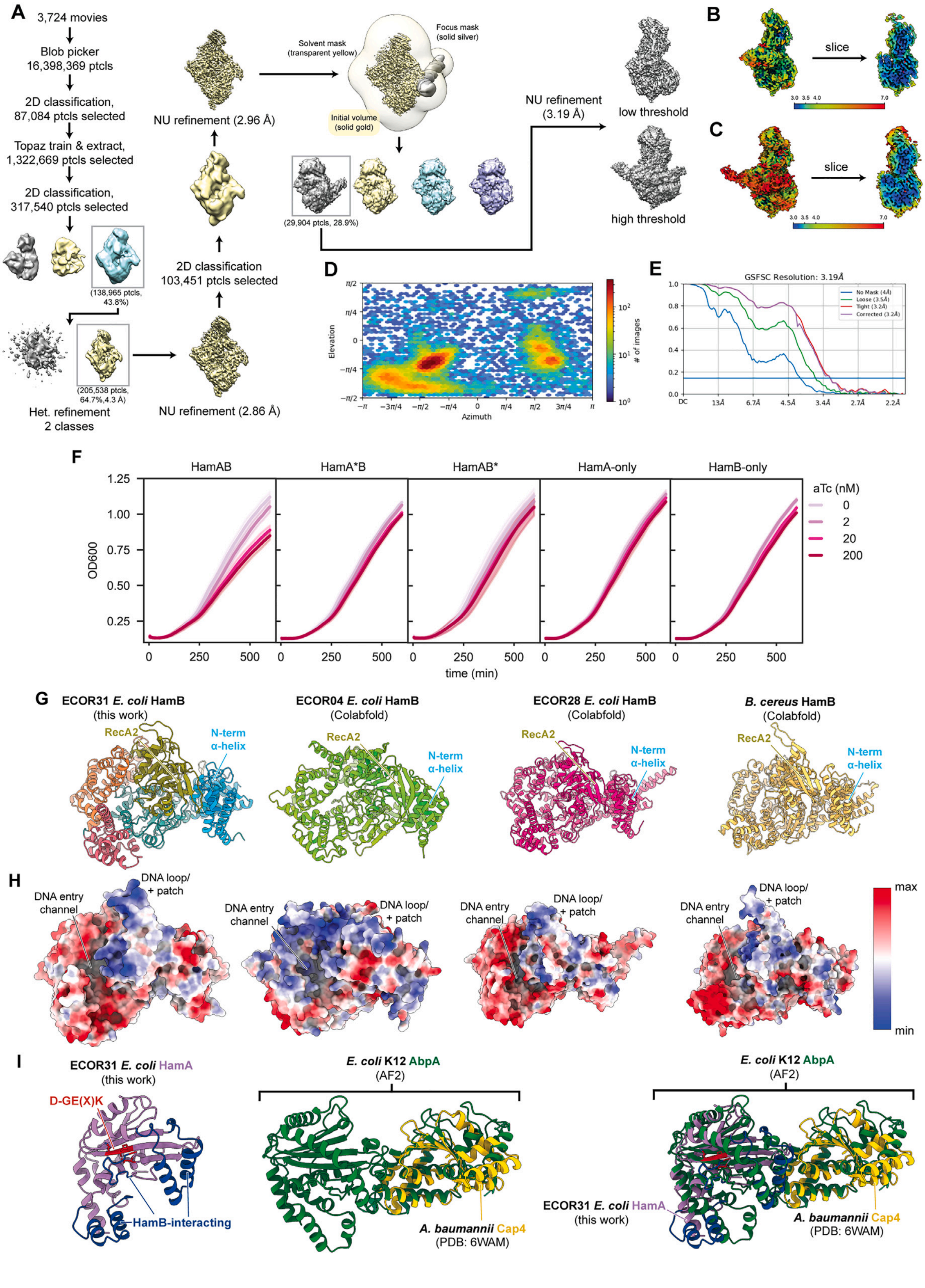

(legend on next page)

CellPress
OPEN ACCESS

Figure S7. Cryo-EM structure of a HamA^{E138A,K140A}B-plasmid DNA complex, related to Figure 7

(A) Particle picking, classification, and refinement strategy to generate the final HamA^{E138A,K140A}B-plasmid DNA density.
(B and C) Final densities at low (B) and high (C) thresholds colored by local resolution. An inner surface slice is shown to the right.
(D) Orientation distribution of the final particle set.
(E) Gold-standard FSC curve.
(F) Toxicity from different levels of Hachiman induction (aTc). All growth curves performed in biological triplicate. The mean and standard deviation of each condition are plotted.
(G) Comparison of ECOR31 HamB from this study with ECOR04 HamB, ECOR28 HamB, and *Bacillus cereus* HamB colabfold predictions.
(H) Electrostatic surface potential representations of the structures from (F), in the same orientation and scale. The DNA entry channel and RecA2 DNA loop/positively charged patch are indicated.
(I) HamA and Cap4 structural superimpositions with AbpA.
